# Measuring vulnerability among female sex workers in India using a multidimensional framework

**DOI:** 10.1371/journal.pone.0204055

**Published:** 2018-09-25

**Authors:** Bidhubhusan Mahapatra, Ruchira Bhattacharya, Yamini Atmavilas, Niranjan Saggurti

**Affiliations:** 1 Population Council, Zone 5A, India Habitat Center, New Delhi, India; 2 National Institute of Rural Development and Panchayati Raj, Rajendranagar Mandal, Hyderabad, India; 3 Bill & Melinda Gates Foundation, The Capital Court, New Delhi, India; Jiangsu Provincial Center for Disease Control and Prevention, CHINA

## Abstract

Measuring vulnerability and identifying determinants of vulnerability are key to designing interventions for marginalized groups like sex workers. The current study introduces a new approach of measuring vulnerability among female sex workers (FSWs) by adopting a multidimensional poverty measurement framework. A multidimensional vulnerability index was created from four dimensions and 16 indicators using a dual cut-off approach. The study found that 55% of FSWs were multidimensionally vulnerable with 48% of intensity in vulnerability. The overall value of multidimensional vulnerability index was 0.265. FSWs in Maharashtra were most vulnerable (82%). Lack of financial security contributed mostly to FSWs’ vulnerability. Further, compared to less vulnerable FSWs, multidimensionally vulnerable ones were more to engage in behaviors that put them at risk such as inconsistent use of condoms with clients, alcohol consumption, engaging in anal sex with clients and experiencing sexually transmitted infections. Findings suggest that structural, social and financial vulnerabilities of FSWs need to be addressed concurrently.

## Introduction

Vulnerability among female sex workers (FSWs) is a widely researched area [1–5]; however, the definition and use of the term “vulnerability” vary considerably with a clear agreement that HIV vulnerability is different from HIV risk [6, 7]. While HIV risk refers to behaviors or situations that directly increase the probability of HIV transmission, HIV vulnerability refers to the context governing individual’s ability to prevent oneself from getting HIV infection [7]. These vulnerabilities can be in various forms ranging from personal attributes to financial, environmental and cultural norms including stigma and discrimination at various levels. Empirical research has shown that vulnerabilities increase FSWs’ HIV risk-taking behaviors including experience of violence, poor negotiation skills to use condom, inability to refuse clients for sex and lack of access to HIV prevention services [8–11]. Given the HIV epidemic in India is a concentrated one and FSWs remain as one of core source of transmission, the current HIV prevention efforts directed towards FSWs need to continue. However, to make the on-going prevention efforts more successful and sustainable, there is a need to address the different vulnerabilities faced by FSWs and hence, enhancing their ability to adopt safe free behavior without any compulsion or fear. The first step in this direction is identifying vulnerable FSWs and measuring the degree of vulnerability faced by them.

Vulnerability measurement revolves around two frameworks: unidimensional and multidimensional. A review of existing literature suggests that most researchers have adopted a unidimensional framework to identify vulnerability [7, 12–14]. Only two Indian studies have considered the multi-dimensional nature of vulnerability by creating a composite index of vulnerability. One of these studies considered three factors (solicitation in street-based setting, experience of violence and financial debt) to create a composite measure of vulnerability [15]. The other study considered six factors (literacy, duration in sex work, mobility for sex work, an additional source of income other than sex work, currently being in debt, and having children) to create an index of vulnerability. However, both these studies were limited by the number of indicators considered for creating the index, a fact which has been acknowledged by the authors themselves.

While multidimensional approach is more comprehensive than unidimensional approach, the former one needs to be aggregated appropriately to estimate vulnerability accurately. In multidimensional approach, vulnerability can be defined in two ways: union approach (vulnerability in any indicator) and interaction approach (vulnerability in all indicators). While union approach over-estimates vulnerability, interaction approach under-estimates. Therefore, this paper addresses this issue of aggregation by employing a “dual cut-off” approach in a multidimensional framework. Moreover, we estimate the intensity of vulnerability in addition to incidence of vulnerability and estimate contribution of each indicator to the overall vulnerability enabling policymakers to identify points of intervention. This study further examines the effect of vulnerability on FSWs’ HIV-related sexual risk behavior.

## Materials and methods

### Study sites

The study was conducted in four southern (Andhra Pradesh, Telangana, Karnataka, Tamil Nadu) and one western (Maharashtra) Indian states. The states of Andhra Pradesh and Telangana were considered as one sampling domain in this study. These states are historically known for high HIV prevalence, and large number of FSWs. The HIV prevalence among FSWs in Andhra Pradesh/Telangana and Karnataka is around 6% each, 7% in Maharashtra, 1% in Tamil Nadu [16]. The living condition of FSWs in these areas was one of the poorest with a large proportion of FSWs being exposed to harsh socio-environmental stress such as lack of proper health care and security. Nearly half the FSWs in Maharashtra, two-thirds in Andhra Pradesh and Telangana, one-fifth in Karnataka and two-fifth in Tamil Nadu depended solely on earnings from sex work to make a living [16]. Moreover, a considerable proportion of FSWs in these states reported experience of general stigma (ranges from 21–42%, national level: 27%) and discrimination at health facility (ranges from 15%-32%, national level: 21%) [16]. Also, a considerable higher proportion of FSWs from these states experienced physical and sexual violence as compared to the national average.

### Data and study design

Data were drawn from a cross-sectional survey conducted in 2015 among 4098 FSWs across five Indian states [17]. FSWs who were 18 years or older and had sex in exchange for cash or kind in the month preceding the survey were included in the survey. The study was conducted among 4098 FSWs who were selected using a three-stage sampling procedure to ensure appropriate geographical representation. The detailed sampling process is published elsewhere [18]. All interviews were conducted by trained female investigators with verbal and written skills in the local language of each state. A structured survey schedule was used for collecting data using face-to-face interview techniques.

### Ethics statement

The institutional review boards (IRBs) of Population Council and Sahara, center for residential care and rehabilitation, reviewed and approved the study procedure and tools. In accordance with the protocol, written consent was obtained from all respondents prior to their participation in the survey. The interviewers read the complete script of the consent form to the respondent and explained if there was any doubt about any aspects of the survey. Participants who could not read and write, the consent process was explained in the presence of a witness (either program staff or fellow sex worker) and verbal consent was taken for such participants. Both the IRBs approved the oral consent in the presence of a witness for respondents who could not read and write. All the interviews were held in a private location specifically hired for the survey, or in a location convenient to the study participants.

### Measures

#### Multidimensional vulnerability index (MVI)

We used the Alkire-Foster (AF) method [19, 20] to create an index, MVI. Mathematically, the MVI is computed using the following equations:

MVI=H*A

Where,
H is the vulnerability headcount ratio and refers to the proportion of FSWs who are multidimensionally vulnerable;
A is the intensity of vulnerability and refers to the average weighted vulnerability experienced by the multidimensionally vulnerable and computed as a division of censored vulnerability score upon number of people who are multidimensionally vulnerable.
and MVI denotes the total vulnerability possible which is experienced by the vulnerable FSWs. The value of MVI ranges from 0 to 1; 0 indicating nobody is multidimensional vulnerable and 1 indicating everyone vulnerable in the study population.

Key steps for computing MVI involves selection of dimensions and indicators of vulnerability, defining indicator level vulnerability cutoffs to identify when an FSW is vulnerable in an indicator, choice of weights to ensure different indicators are accounted for their relative importance, and identifying a second-stage cutoff to determine when an FSW to be considered as multidimensionally vulnerable.

#### Selection of dimensions and indicators and identifying vulnerability cut-offs for indicators

We used four dimensions and 16 indicators to capture different aspects of vulnerability. Table 1 provides the details of each dimension and indicators as well as the rationale behind considering them as indicators of vulnerability. The four dimensions of vulnerability considered are personal attributes, financial security, social protection and social support and network. Personal attributes aim to capture disempowering attributes or factors that put lives of FSWs at a greater risk than their counterparts and include four indicators. The dimension of financial security contains five indicators reflecting economic situation of FSWs. Similarly, dimension on social protection includes four indicators reflecting FSWs’ access to various social schemes, food security, and awareness of basic rights. The dimension of social support and network has three indicators, each reflecting depth of FSWs’ social network. Lack of social network can exclude FSWs from social gatherings and jeopardize their ability to demand any need. Given that the validity and stability of the index rest upon the intuition behind these indicators and their actual relevance for the study population, all the indicators were thoroughly reviewed, discussed and assessed for their programmatic and scientific relevance. Each of these indicators was defined in a way to generate dichotomous variables with “1” indicating vulnerable condition; else “0” indicating non-vulnerability.

**Table 1:** Dimensions, indicators, and weights used in computing multi-dimensional vulnerability index among FSWs, India.

Dimension	Components	Indicators: Vulnerable if…/ justification	% of FSWs	Weight
Personal attributes	Age	*Age is < 25 years*: Young FSWs <25 years old) are at increased risk of getting HIV infection due to a higher client volume and low negotiation power [33–35].	9.9	1/4*1/4 = 0.063
	Place of solicitation	*Soliciting in street*: FSWs soliciting in streets are at increased risk of HIV infection due to their social status and working conditions on the street. Also, they lack access to safety or sanitary measures and social network [7, 33, 36].	23.9	1/4*1/4 = 0.063
	Degree of dependency	*More than two dependents on FSWs’ income*: FSWs with large Household size with more number of dependents are at financial stress and may indulge in risk behavior [27, 37].	64.0	1/4*1/4 = 0.063
	Mobility	*FSW moves frequently from one city to other for sex work or have moved to the place of interview for sex work*: For sex workers whose frequency of changing place for sex work is *Monthly or More* (Highly mobile), or those *not a native* of the district where they live and work, community support is low and access to safety or health related services is difficult. They are also exposed to violence [7, 9, 13, 27, 38].	44.4	1/4*1/4 = 0.063
Financial security	Livelihood	*There is no other alternative income source*: Sole dependence on sex work increases the chance of unsafe sexual practices. Financial stress of single livelihood sex workers is also higher than others [2, 6, 27].	47.8	1/4*1/5 = 0.050
	Savings account	*FSW does not have any saving account in either bank or post office*: The risk of low income, and non-access to social transfer or lack of financial security measure is increases without bank account. The financial insecurities increase likelihood of risky behavior [6, 28].	31.6	1/4*1/5 = 0.050
	Savings or investment	*FSW has neither saving nor made any investment in either land*, *gold or house*: FSWs without any asset or investment are prone to higher income volatility and resultantly may depend on loan or risky sexual behavior as coping mechanism [6, 28].	61.3	1/4*1/5 = 0.050
	Indebtedness	*FSW has taken a loan from an informal source in last 12 months*: Taking a loan from informal sources such as money lender, and friends makes FSW financially vulnerable and prone to resort to unsafe sexual behavior in crisis [6, 27, 30].	20.0	1/4*1/5 = 0.050
	Insurance	*FSW does not have any health*, *life or accident insurance for herself*: Lack of insurance makes FSWs exposed to income or consumption shocks and leave them with no mechanism to overcome the crisis [6, 28].	86.9	1/4*1/5 = 0.050
Social protection	Citizen identity card	*FSW has no unique identification (Aadhar) or voter identity card and does not have permanent account number (PAN) card*: Lack of identity card or citizenship hinders FSWs access to any socio-financial entitlement, gainful employment and even a normal life [6, 39].	5.3	1/4*1/4 = 0.063
	Food security	*FSW reported food insecurity in last 6 months*. *Food insecurity refers to either "eating less than needed" or "smaller meal" or "not eating at all" in at least one occasion in last six months*: Food insecurity is marker of extreme poverty and makes FSW vulnerable to risky behavior and may create compulsion of taking loan [6, 26].	16.2	1/4*1/4 = 0.063
	Ration card	*FSW does not possess any ration card*: Lack of ration card hampers FSWs receive food entitlements and increase their expenditure bargain [6].	20.3	1/4*1/4 = 0.063
	Awareness of about rights	*FSW has not received any training on legal education and rights*: Lack of legal training makes FSWs vulnerable to legal exploitation and increases their dependency on CO or NGO [14, 32].	63.3	1/4*1/4 = 0.063
Social network	Membership in community-led organization (CO)	*FSW is not a member of FSW CO*: Not being a member of CO exposes FSWs to safety risks, bars their access to targeted interventions and other safety products and lowers their social capital [3, 10, 24, 40].	22.9	1/4*1/3 = 0.083
	Access to HIV prevention services	*FSW has not received any HIV prevention services in last 12 months*: Lack of STI/HIV service makes FSWs vulnerable to risky behavior and HIV infection [3, 10].	26.9	1/4*1/3 = 0.083
	Community coherence	*CO members did not come together to help FSW at the time of crisis in past 6 months*: Lack of community coherence dilutes the purpose of collectivization. Strong bonding between community members provides confidence and security to the FSWs [24, 41].	51.8	1/4*1/3 = 0.083

#### Weighting of indicators

All dimensions and indicators within each dimension were assigned equal weights. This means each dimension received a weight of 1/4. Weight for an indicator within a dimension was calculated as reciprocal of number of indicators in the dimension multiplied by number of dimensions. For example, if there were five indicators in one dimension, then weight for indicator would be 1/4*1/5 = 0.05. The weights were multiplied with each indicator (coded as 0 and 1) to generated weighted indicator values. The weighted values of all indicators were summed to generate a weighted vulnerability score.

#### Identifying multidimensionally vulnerable FSWs

**Fig 1:**
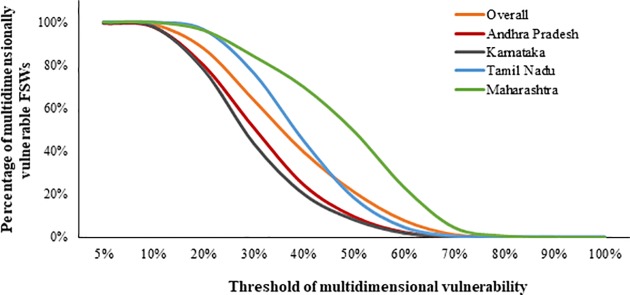
Proportion of multidimensionally vulnerable FSWs for different threshold values of multidimensional vulnerability.

FSWs whose weighted scores were above a certain threshold level *k* were considered multidimensionally vulnerable. The selection of the threshold value is a normative decision considering the indicators included and how important they are in defining vulnerability [19, 21]. This study has four dimensions, thus having a threshold value of *k* = 0.25 would qualify as multidimensional vulnerable. However, after a series of robustness test, we choose to have a threshold of 0.33 which means an FSW is multidimensionally vulnerable if she is vulnerable in more than one-third of weighted vulnerability score. We checked the robustness of the level by comparing the state-specific proportion of multidimensionally vulnerable FSWs (Fig 1) and MVI values (Table 2) at different threshold values. If they hold a consistent pattern, they were considered as robust. The consistency was checked using Kendal Tau-b coefficient of rank correlation which compared the ranking of states for MVI estimates at different threshold values [19]. More detailed steps of calculation of multidimensional indexing and its properties can be found elsewhere [19, 20].

**Table 2:** Multidimensional vulnerability index (MVI) estimates for different threshold values (*k*) by state to test the robustness of estimate.

	*k* = 0.25	*k* = 0.33	*k* = 0.40	*k* = 0.50
Andhra Pradesh	0.246	0.177	0.122	0.055
Karnataka	0.226	0.152	0.099	0.046
Tamil Nadu	0.370	0.296	0.222	0.103
Maharashtra	0.463	0.436	0.394	0.301

### HIV-related sexual risk factors

HIV-related sexual risk factors were assessed in terms of inconsistent condom use with clients, alcohol consumption, anal sex in last 12 months, ability to negotiate for condom use with clients and experience (self-reported) of sexually transmitted infections (STIs) in past six months. FSWs were asked separately about the frequency of condom use with occasional and regular clients in past one month with response options of ‘always’, ‘most of the time’, ‘sometimes’ or ‘never’. FSWs who used a condom “always” with both clients were considered consistent condom user; otherwise considered inconsistent user (S1 Appendix). Ability to negotiate for condom use was derived from a question on any instance in past six months when the FSW wanted to use a condom but could not do it with response categories “no” and “yes”. Those responded “yes” were considered to have poor ability to negotiate; else considered to have the ability to negotiate for condom use. To determine the experience of STI symptoms, participants were asked whether they had experienced any of the following symptoms in the six months prior to the survey: ulcers/sores in the genital area, swelling in the groin area, and frequent painful urination. Those responding “yes” to any of these symptoms were classified as having experienced STI symptoms (Yes = 1, No = 0). FSWs’ treatment seeking behavior in last 12 months, engaging in anal sex in last 12 months, alcohol consumption, and current use of any family planning method was assessed using single-item questions. Information on socio-demographic variables like marital status (currently married, never married and formerly married), education (no formal education, formal education), and duration in sex work (continuous) was assessed using single item questions.

### Statistical analyses

Univariate analysis was used to present the percent of FSWs who are vulnerable in different indicators. Bivariate analysis was used to present the levels of different HIV-related sexual risk factors by the multidimensional vulnerability. A series of multiple logistic regression models were fitted to examine the effect of multidimensional vulnerability on different HIV-related sexual risk behaviors. In each of these logistic regressions, multidimensional vulnerability was the key independent variable whereas socio-demographic factors were used as covariates and indicators on HIV-related sexual risk behaviors were dependent variables. Results were presented in the form of percentages, adjusted odds ratios (AOR) and their corresponding 95% confidence interval (CI). All the analyses were performed using STATA 13.1 (StataCorp., TX, USA).

## Results

Surveyed FSWs were, on an average, 34 years and practicing sex work for about seven years. A little above two-fifths (42%) of FSWs were having no education, and about three-fifths (62%) were currently married (Data not shown).

### Multidimensional vulnerability

#### Intensity and headcount of multidimensional vulnerability

More than half (55%) of FSWs were multidimensionally vulnerable (Table 3). The proportion of FSWs multidimensionally vulnerable was highest in Maharashtra (82%) and least in Karnataka (35%). The intensity of vulnerability was 48%, which suggests that multidimensionally FSWs were vulnerable in nearly half of the weighted vulnerable indicators. The intensity of vulnerability was highest in Maharashtra (53%). In the other three states, intensity of vulnerability among vulnerable FSWs was almost similar. The overall MVI was 0.265 with the highest level in Maharashtra (0.436) and least in Karnataka (0.152).

**Table 3:** Estimates of multidimensional vulnerability measures at threshold value of *k* = 0.33 among female sex workers in India.

Measures	Andhra Pradesh	Karnataka	Tamil Nadu	Maharashtra	Overall
Percentage of FSWs who are multidimensionally vulnerable	40.1	34.9	65.0	81.6	55.3
Average intensity of vulnerability	44.1	43.7	45.6	53.4	47.8
Multidimensional vulnerability index (MVI)	0.177	0.152	0.296	0.436	0.265
Percentage of FSWs who are severely vulnerable^€^	9.7	8.1	18.2	49.3	21.1

^€^Severely vulnerable if vulnerable in 50% or more weighted indicators.

### Contribution of different factors to multidimensional vulnerability

**Fig 2:**
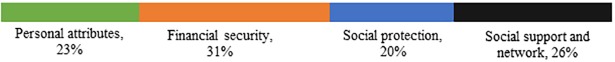
Contribution of different dimensions to overall vulnerability among female sex workers in India.

The dimension of financial security contributed most to FSWs’ vulnerability (31%), followed by social support and network dimension (26%) (Fig 2). Personal attributes contributed 23% to overall vulnerability, whereas contribution of social protection was only 20%. In terms of individual indicators, the highest contribution to overall vulnerability was from lack of community coherence (12%), followed by lack of any insurance (10%), no exposure to legal awareness training (10%) and having two or more dependents (10%) (Table 4). Lack of savings or investment contributed about 8% to overall vulnerability.

**Table 4:** Intensity of vulnerability for different indicators and their contribution to overall vulnerability among female sex workers in India.

Indicators	% contribution to overall vulnerability	% vulnerable among multidimensionally vulnerable
**Personal attributes**		
Age	1.9	8.1
Place of solicitation	3.5	14.7
Degree of dependency	9.6	40.5
Mobility	7.7	32.6
**Financial security**		
Livelihood	6.2	32.9
Savings account	4.9	25.8
Savings or investment	8.2	43.2
Indebtedness	1.9	10.0
Insurance	10.0	52.7
**Social protection**		
Citizen identity card	3.4	14.4
Food security	2.7	11.3
Ration Card	4.4	18.5
Legal rights	10.0	42.2
**Social support and network**		
Membership in CO	6.7	21.2
Access to HIV prevention services	7.0	22.4
Community coherence	12.1	38.5

The major contributors to vulnerability was also prevalent in the study population as indicated by the proportion of vulnerable FSWs for different indicators among multidimensionally vulnerable sub-sample (Table 4). For example, A little more than half (53%) did not possess any insurance, about two-fifths had no financial savings or investments (43%) and had two or more dependents (41%) and had no exposure to legal awareness trainings (42%) and lacked community coherence (39%). The proportion of FSWs in these indicators also remained high in the overall study population as can be seen in Table 1. For example, FSWs who had no insurance was 87% in the overall sample and reduced by 30% when the sample is restricted to multidimensionally vulnerable FSWs.

### Association between multidimensional vulnerability and HIV-related sexual risk factors

Multidimensionally vulnerable FSWs were more likely to report negative health behaviors compared to those multidimensionally non-vulnerable ones (Table 5). For example, inconsistent use of condoms with clients in past one month was almost twice higher among multidimensionally vulnerable FSWs than non-vulnerable ones (AOR: 1.75, 95% CI: 1.35–2.26). Similarly, multidimensional vulnerability was positively associated with inability to use condom even when FSWs wanted to use it (AOR: 1.58, 95% CI: 1.22–2.04), practice of anal sex (AOR: 1.42, 95% CI: 1.12–1.79) and alcohol consumption (AOR: 1.51, 95% CI: 1.29–1.77). FSWs who were multidimensionally vulnerable were also less likely to go for HIV testing in past two years and STI check-up and counseling services in past 12 months.

**Table 5:** Unadjusted percent and adjusted odds ratio with corresponding 95% confidence interval predicting HIV-related sexual risk behaviors with multidimensionally vulnerable as key predictor variable adjusted for socio-demographic variables among female sex workers, India (N = 4098).

Indicators	Multidimensionally vulnerable		AOR (95% CI)^€^
	No	Yes	
Inconsistent condom-use with clients in last one month	5.6	11.2	1.75 (1.35–2.26)
Wanted to use condom but could not use in past 6 months	6.0	10.9	1.58 (1.22–2.04)
Had anal sex with clients in last 12 months	7.7	14.1	1.42 (1.12–1.79)
Experienced self-reported sexual transmitted infections (STI) in past 6 months	13.2	16.4	1.17 (0.97–1.42)
Currently consuming alcohol	20.7	30.6	1.51 (1.29–1.77)
Did not go for STI check-up and counselling services in last 12 months	34.4	60.4	2.69 (2.34–3.10)
Tested for HIV < 3 times in last two years	16.1	19.2	1.39 (1.16–1.66)
Currently not using any family planning method	32.0	37.6	1.35 (1.17–1.57)

^€^AOR: Adjusted Odds Ratio; CI: Confidence Interval

Note: Multiple logistic regression models were adjusted for duration in sex work, educational status, marital status and state where interview was conducted.

## Discussion

Measurement of vulnerability and identifying the context leading to vulnerability is important for strategic planning for any intervention. FSWs in India face multiple vulnerabilities by the nature of their profession; hence, identification and measurement of vulnerability in a robust way is necessary for reaching the vulnerable population. As the extent and nature of vulnerabilities vary considerably depending on the definition, measurement approaches and interpretation [7], it can be perplexing at-times to stakeholders and implementers. In this context, this study suggests a more comprehensive way of measuring vulnerability among FSWs by adopting an approach used mostly to measure poverty. The approach has not only helped in integrating the multiple dimensions and indicators of vulnerability but also provided the intensity of vulnerability alongside the prevalence of vulnerability [22]. Moreover, this approach is statistically more superior compared to other approaches of computing index as has been demonstrated in the computation of multidimensional poverty index [23]. The study found that a large share (55%) of FSWs in the study area were multidimensionally vulnerable. FSWs in Maharashtra were most vulnerable (82%) and least in Karnataka (35%). Lack of financial security contributed mostly to FSWs’ vulnerability. Specifically, lack of possession of any insurance scheme, and lack of savings or investment in gold, property or land contributed mostly to FSWs’ vulnerability.

Multidimensionally vulnerable FSWs were also more likely to be at increased HIV risk and less likely to go for STI check-up and HIV testing than those multidimensionally non-vulnerable. This reflects the robustness of our approach in identifying vulnerable FSWs. Moreover, it indicates that reducing vulnerabilities among FSWs would eventually contribute to a reduction in their risk-taking behavior. This is in-line with the past research in India which suggests that addressing vulnerabilities among sex workers could lead to increase in their safe sex behaviors [4, 6, 7, 10, 24, 25]. While sex workers enter into sex work due to increasing financial burden, trafficking and coercion, their vulnerabilities increase after getting into sex work due to the non-conducive policies and institutional arrangements. In a patriarchal society, their power of negotiation with clients (for money, type of sex and use of condoms) gets weakened to the social and financial vulnerabilities they are facing simultaneously. Therefore, addressing these vulnerabilities will empower FSWs and hence, the ability to negotiate for sex will increase [5, 9, 24, 26, 27].

The study also found a negative relationship between vulnerability and STI check-up and HIV testing which is contradicting the earlier study in Mumbai, India which suggested that high vulnerability of FSWs (though measured differently than this study) linked to higher service uptake. The difference can be due to the fact the earlier study was conducted among individuals who were part of the targeted HIV prevention intervention and the intervention prioritized providing services to the highly vulnerable ones [7]. Therefore, the earlier study showed a positive relationship between vulnerability and service uptake.

The vulnerability among FSWs was primarily owing to lack of access to financial security. It is well-known fact that FSWs in India face challenges of a different nature compared to women in general population to access the financial services or take benefits of social schemes [5]. The clandestine nature of sex work and stigma associated with sex work practice has limited FSWs’ access to different government services including health and social services [28]. Moreover, the fact that majority of sex workers enter the industry due to financial burden and hence, addressing financial and social vulnerabilities of sex workers is utmost important to improve the ability to negotiate with clients, brothel owners and pimps [27, 29]. Also, evidence suggests that FSWs with high financial vulnerability and lack of access to social entitlements are more likely to engage in risky sexual behavior [2, 6, 10, 26–28, 30]. Therefore, addressing the financial needs and improving financial inclusion and capability among FSWs will not only address vulnerabilities but also improve the likelihood of safe sex practice among them.

The contribution of individual factors within each dimension varies considerably across dimensions. Within the domain of financial security, lack of insurance contributed most (32%), followed by contribution from lack of savings or investment in gold, land or property (26%), lack of alternative income other than sex work (20%) and not having saving accounts in bank or post office (16%). Loan from an informal source contributed only 6%. While in previous vulnerability research loan has been considered as a key factor [7, 15], our study showed that other economic indicators are more relevant to assess FSWs’ vulnerability than only financial debt. Within the social protection dimension, the contribution to vulnerability was mostly due to lack of awareness on legal rights (49%), followed by lack of possession of ration card (21%) and citizen identity card (17%). Food insufficiency contributed the least (13%) to social protection dimension which again in contrast to previous research which argues for food insufficiency as the key vulnerability. Rather, it is lack of owning a ration card is a more important contributor of vulnerability among FSWs in India.

Most of the existing research has treated indicators of personal attributes as covariates while undertaking analysis involving vulnerabilities of FSWs. In this study, instead of considering them as covariates, we considered them as key factors that contribute to FSWs’ vulnerability. The findings suggested that 23% of the vulnerabilities among FSWs were due to their personal attributes. The indicators within personal attributes that contributed most is degree of dependency (42%) and being mobile/migrant for sex work (34%). The role of degree of dependency on FSWs’ vulnerability has been highlighted in prior research also ^25^. While previous research in India suggests that place of solicitation is the key factor that decides FSW’s vulnerability and exposure to risk, we found that contribution from street-based solicitation is considerably low (4% to overall vulnerability). This is primarily due to consideration of multi-dimensional framework as against a unidimensional approach considered by previous studies. Similar to the dimension of personal attributes, the absence of social support and network contributed about one-quarter to overall vulnerability level. The indicator on social coherence (measured as community members did not come together to help FSW at the time of crisis) contributed most to the social support and network dimension. Given the increasing importance of community mobilization interventions among FSWs, this finding reiterates the need for such intervention.

The study’s findings should be interpreted in the light of following limitations. First, there may be social desirability bias in some of the behavioral measures such as condom use due to their prior exposure to HIV prevention programs. Second, the relationship between vulnerability and HIV-related sexual risk factors are associative in nature given that the analysis was conducted on a cross-sectional data. Like the multidimensional poverty measurement, the multidimensional vulnerability measure is based on certain normative decisions. One can always argue the rationale behind the inclusion or removal of certain indicators or categorization of an indicator within a specific dimension. We completely acknowledge the possibility of refinement in the measurement process and see this is one of the first steps in adopting a multidimensional framework to measure vulnerability.

The study findings have important policy implications for programs working with sex workers. First, vulnerabilities of FSWs should not be seen from the context of sex work alone, rather, the structural, social and financial vulnerabilities need to be addressed to provide individuals a safe and enabling environment. Second, intervention programs need to adopt a multi-pronged strategy to address multidimensional vulnerabilities given that the vulnerabilities vary in nature and scale. The intensity of vulnerability and their contribution to overall vulnerability level can be a first step in developing intervention strategies to reduce vulnerability. The interventions should specifically design programs to address financial vulnerabilities by facilitating access to formal financial services and helping them to increase their saving habits and make investments in different movable and immovable assets. Some of the notable interventions in India are Songachhi project in Kolkata, and the Pragati intervention in Bangalore, where addressing the financial vulnerabilities have been at the forefront which has led to improved HIV risk behaviors [26, 31]. Lack of awareness of legal rights is another area which needs to address as with proper knowledge on different rights, they may be more resilient to exploitation [32]. In addition, given the increasing emphasis of providing insurance to all individuals by Government of India, and lack of insurance a key contributor to vulnerability, interventions working FSWs should devise strategies that help sex workers’ getting access to insurance. Finally, the effort on building community collectivization should continue as it can reduce the vulnerabilities among FSWs. In conclusion, this study demonstrated how a multidimensional approach to measuring vulnerabilities among FSWs may help in identifying a range of factors that can be prioritized by interventions. Based on this study the importance of measuring vulnerabilities from an aggregate framework is emphasized because a vulnerability which may come out as important in a single-dimensional study may prove to be less important when putting in a multidimensional frame. Further, the study showed positive associations between vulnerability and HIV-related sexual risk factors suggesting that addressing vulnerabilities of FSWs would eventually lead to better HIV risk behavior among them.

## Supporting information

S1 Appendix Questionnaire for behavioral data collection using the member engagement and communication tool. (DOCX)
