# Supplementary material for: Measuring vulnerability among female sex workers in India using a multidimensional framework
Source: PLoS One. 2018 Sep 25;13(9):e0204055. doi: 10.1371/journal.pone.0204055 (PMC6155505; doi:10.1371/journal.pone.0204055)
Supplement: S1 Appendix — (DOCX) [file pone.0204055.s001.docx]

**Evaluation of Avahan Community Mobilization Phase III**

**Female Sex Workers**

Questionnaire for Behavioral Data Collection using the Member Engagement and Communication Tool (MECT)

**Population Council**

**New Delhi, India**

| **INTRODUCTION**  .   1. Greetings (for example: Good Morning/Good Afternoon/Good Evening). 2. Introduce yourself. 3. Emphasize the confidentiality and importance of the responses. 4. Thank the person for having agreed to participate. |
| --- |

**Operational Definition:**

**Female Sex Worker (FSW):** **Woman, aged 18 years or more, who engaged in consensual sex in exchange for money/payment in kind in the last one month.**

**Informed Consent Form for expanded MECT**

**with Female Sex Workers**

**Evaluation of Avahan Community Mobilization Phase III**

*Namaste* (Greetings!). My name is <NAME OF THE INVESTIGATOR> and I am working with <NAME OF THE ORGANIZATION>.

You are invited to take part in an evaluation study. Before you decide whether to participate, you need to understand why the study is being done and what it would involve. Please take the time to read or listen as I read the following information. You may talk to others about this evaluation study if you wish. Please ask me if there is anything that is not clear, or if you would like more information. When all of your questions have been answered and you feel that you understand this study, you will be asked if you wish to participate in the study, and if yes to sign this informed consent form.

**Purpose of the Study**

The purpose of the study is to measure the impact of the Avahan program on reducing structural (e.g. violence from police), economic (e.g. debt) and social (e.g. social schemes and entitlements) barriers, and examine how these changes promote safe sex practices in this state. We are interested in learning from people like you to understand what kind of role the local community organizations are currently playing in promoting access to social entitlements, financial services, and crisis response systems, and the process involved in overcoming challenges.

**Procedures to be followed**

Your name has been selected from the list managed by the local program. We are inviting you to participate in this study because we believe that your responses will help improve the program so that many individuals can benefit. In the interview, you will be asked questions on client solicitation and condom use practices, sexual and reproductive health, access to safety, security, and justice, social protection, and financial security services, utilization of HIV services, and mental health. You will be asked these questions now and, if you consent, we will ask these questions again up to two times over the next four years. Additionally, we will ask your name and contact details for our future reference. This information will be taken exclusively for study purposes and it will not be shared with any one. The interview will take approximately 40-50 minutes in each session.

**Risks**

You may experience some discomfort or risks related to: (a) fear about the loss of confidentiality regarding sensitive information; (b) some of the questions as they may be emotionally sensitive. You do not have to respond to any question that makes you uncomfortable.

**Benefits**

There is no direct benefit to you. An indirect benefit may be in knowing you have participated in an important evaluation study that could help others like you in the future through its findings and programmatic changes.

**Confidentiality**

The information that is collected during this evaluation study will be kept strictly confidential. No one will be told that you have participated in the study. The study team will make every effort to protect your privacy and maintain the confidentiality of all the information that you provide. Your name and contact details will not appear on any of the electronic data or in reports from this study. Your name and contact details will be noted separately in a sheet along with this form will be stored in a locked cupboard at Population Council office and will be used only for study purposes by professional staff.

**Voluntariness**

Your participation in this study is voluntary. If you decide not to participate, you will not lose any existing benefits to which you are entitled. If you agree to participate in this study, you may end your participation at any time without penalty or loss of existing benefits to which you are entitled. If you decide to take part, you are free to refrain from answering any questions. You are free to withdraw at any time without affecting your relationship with the local programs or the community organization that you belong.

**Additional information**

You will never be identified in any presentations or publications based on the results of this evaluation study. We will report the data in an aggregate form in most possible ways.

If you have a concern about any aspect of the study, you should ask to speak to the researchers who will do their best to answer your questions. You may call: Dr. Rajatshuvra Adhikary at this number 011-41743410.

If you have concerns regarding the study or injuries, please contact: Dr. Avina Sarna, at Population Council: 011-24642901 (or) 24642902.

If you want to know about your rights for being participant in the study, please contact: Ms. Siddhi Mankad, Member of the Accountability and Transparency Sub-Committee for Swasti Governing Board, at: +91-9342468350.

**Subject Statement:** I have read the Informed Consent for this study. I have received an explanation of the planned study, procedures, risks and benefits and privacy of my personal information. I agree to take part in this study. I understand that my participation in this study is voluntary.

**Your name: ___________________________________________**

**Your signature: __________________________________Date:____________**

**Instructions to investigator:** Agreed to participate, but not willing to sign this form (or) cannot sign, then check mark below.

***( ) Oral consent given***

Interviewer’s statement: I, the undersigned, have explained to the participant in a language participant understands the procedures to be followed in the study and the risks and benefits involved.

_________________ _________________________ ______________________

Date Name of Investigator Signature of Investigator

**Investigator or person who conducted Informed Consent discussion:** I confirm that I have personally explained the nature and extent of the planned study, study procedures, potential risks and benefits, and confidentiality of personal information.

**Name of person obtaining consent:______________________________________**

**Signature of person obtaining consent:____________________Date:____________**

**Respondents Contact details:**

Village/Street name: ___________________________________________

Mandal/city name: ____________________________________________

District name: ________________________________________________

Organization name running TI: ___________________________________

FW/ ORW name: ______________________________________________

FW/ ORW contact Telephone number: _________________

Respondent’s Name (Mention the alias name in bracket)________________

Telephone (mobile) number of the respondent: _______________________

Alternate contact number of the respondent__________________________

Name of the close friend – 1 ______________________________________

Contact number of the close friend – 1______________________________

Name of the close friend – 2______________________________________

Contact number of the close friend – 2______________________________

Respondent ID (as in questionnaire):________________________________

| **Member Engagement and Communication Tool** | | | | | | | | | | | | | | | | | | | | | | | | | | | | | | | | | | | | | | | | | | | | | | | | | | | |  |  |  |  |  |  |  |  |  |  |  |  |  |  |  |  |  |  |  |  |  |  |  |  |  |  |  |  |  |  |  |  |  |  |  |  |  |  |  |  |  |  |  |  |  |  |  |  |  |  |  |  |  |  |  |  |  |  |  |  |  |  |  |  |  |  |  |  |  |  |  |
| --- | --- | --- | --- | --- | --- | --- | --- | --- | --- | --- | --- | --- | --- | --- | --- | --- | --- | --- | --- | --- | --- | --- | --- | --- | --- | --- | --- | --- | --- | --- | --- | --- | --- | --- | --- | --- | --- | --- | --- | --- | --- | --- | --- | --- | --- | --- | --- | --- | --- | --- | --- | --- | --- | --- | --- | --- | --- | --- | --- | --- | --- | --- | --- | --- | --- | --- | --- | --- | --- | --- | --- | --- | --- | --- | --- | --- | --- | --- | --- | --- | --- | --- | --- | --- | --- | --- | --- | --- | --- | --- | --- | --- | --- | --- | --- | --- | --- | --- | --- | --- | --- | --- | --- | --- | --- | --- | --- | --- | --- | --- | --- | --- | --- | --- | --- | --- | --- | --- | --- | --- | --- | --- |
|  | | | | | | | | | | | | | | | | | | | | | | | | | | | | | | | | | | | | | | | | | | | | | | | | | | | |  | | | | | | | | | | | | | | | | | | | | | | | | | | | |  | | | | | | | | | | | | | | | | | | | | |  | | | | | | | | | | | | | | |  | | | | | | |
|  | | | | | | | | | | | | | | | | | | | | | | | | | | | | | | | | | | | | | | | | | | | | | | | | | | | | Questionnaire ID:  State CO FW Respondent | | | | | | | | | | | | | | | | | | | | | | | | | | | | | | | | | | | | | | | | | | | | | | | | | | | | | | | | | | | | | | | | | | | | | | |
| **Name of the CO: …………………………………………………………………………………………………………………… District Name: ……………………………………** | | | | | | | | | | | | | | | | | | | | | | | | | | | | | | | | | | | | | | | | | | | | | | | | | | | | | | | | | | | | | | | | | | | | | | | | | | | | | | | | | | | | | | | | | | | | | | | | | | | | | | | | | | | | | | | | | | | | | | | | | | |
| ***Section 1 : Profile of the Respondent*** | | | | | | | | | | | | | | | | | | | | | | | | | | | | | | | | | | | | | | | | | | | | | | | | | | | | | | | | | | | | | | | | | | | | | | | | | | | | | | | | | | | | | | | | | | | | | | | | | | | | | | | | | | | | | | | | | | | | | | | | | | |
| 1.6 | Dominant Typology: | | | | | | | | | | Home based | | | | | | | | | | Lodge based | | | | | | | | | | | | | Brothel based | | | | | | | | | | | | | | | Tamasha based | | | | | | | | | | | | | | | Street based | | | | | | | | | | | | | | | | | | | Devdasi | | | | | | | | | | | | Others ( Specify) | | | | | | | | | | | | | | | | | | | | | | | | | | | |
| 1.7 | Where do you stay | | | | | | | | | | | | | | | | | | | | | | | | | | | | | | | | | | | | | | | | | | | | | | | | | | | | | | | | | | | | | | | | | | Home | | | | | | | | | | | | | | | | | | | | | | | Hut | | | | | | | | | | | | | | | | | | | | | | | | Street | | | | | | | | |
| 1.8 | Age of the respondent (In completed years) | | | | | | | | | | | | | | | | | | | | | | | | | | | | | | | | | | | | | | | | | | | | | | | | | | | | | | | | | | | | | | | | | | | |  | | | | | | | | | | | | | | | | | | | | | | | | | | | | | | | | | | | | | | | | | | | | | | | | | | | | | |
| 1.9 | What is the highest standard or class / year of college you completed? | | | | | | | | | | | | | | | | | | | | | | | | | | | | | | | | | | | | | | | | | | | | | | | | | | | | | | | | | | | | | | | | | | | |  | | | | | | | | | | | | | | | | | | | | | | | | | | | | | | | | | | | | | | | | | | | | | | | | | | | | | |
| 1.9A | Are you native of this district? | | | | | | | | | | | | | | | | | | | | | | | | | | | | | | | | | | | | | | | | | | | | | | | | | | | | | | | | | | | | | | | | | | | | Yes  No | | | | | | | | | | | | | | | 1  2 | | | | | | | | | | | | | |  | | | | | | | | | | | | | | | | | | | | | | | | |
| 1.10 | Language abilities | | | | | | | | | | | | | | | | | | | | | | | | | | | | | | | | | | | | | | | | | | | | | | | | | | | | | | | | | | | | | | | | | | | | | | | | | | | | | | | | | | | | | | | | | | | | | | | | | | | | | | | | | | | | | | | | | | | | | | | | | |
| Circle the mother tongue (Circle one only) | | | | | | | | | | | | **Kannada** | | | | | | | | **Telugu** | | | | | | | | | | | **Marathi** | | | | | | | | | | | | | | | | | | | | | | | | | | | | | **Hindi** | | | | | | | | | **Tamil** | | | | | | | | | | | | | | **English** | | | | | | | | | | | | | | | | | | | | | | | | | | **Others (Specify)** | | | | | | | | | | | | | |
| Language Ability | | | | | | | | | | | |  | | | | | | | |  | | | | | | | | | | |  | | | | | | | | | | | | | | | | | | | | | | | | | | | | |  | | | | | | | | |  | | | | | | | | | | | | | |  | | | | | | | | | | | | | | | | | | | | | | | | | |  | | | | | | | | | | | | | |
| **Codes : Speak – A, Read – B, Write - C Language not known-Z** | | | | | | | | | | | | | | | | | | | | | | | | | | | | | | | | | | | | | | | | | | | | | | | | | | | | | | | | | | | | | | | | | | | | | | | | | | | | | | | | | | | | | | | | | | | | | | | | | | | | | | | | | | | | | | | | | | | | | | | | | | |
| 1.11 | What is your marital status? | | | | | | | | | | | | | | | | | Married-1 | | | | | | | | | | Never Maried-2 | | | | | | | | | | | | | | | | | | | | Deserted/Separated/Divorced-3 | | | | | | | | | | | | | | | | | | | | | | | | | | | | | | | | | | | | | Widowed-4 | | | | | | | | | | | | | | | | | | | | Others (specify)-9 | | | | | | | | | | | | | | | | | |
| 1.12 | Currently with whom you are living? | | | | | | | | | | | | | | | | | | | | | | | | | | | | | | | | | | | | | | | | | | | | | | | | | | | | | | | | | | | | | | | | | | | | | | | | | | | | | | | | | | | | | | | | | | | | | | | | | | | | | | | | | | | | | | | | | | | | | | | | | |
| 1.**►1.15** | | | | | | | | 2**►1.15** | | | | | 3 | | | | | | 4 | | | | | | | | | | | | | | | | | | | | | | | 5 | | | | | | | | | | | | | | | | | | | | | | | | | | | | | 6**►1.15** | | | | | | | | | | | | | | | | | | | | | 7**►1.15** | | | | | | | | | | | | | | | | | | | | | | | 77**►1.15** | | | | | | | |
| Living alone | | | | | | | | With Parents | | | | | With Husband | | | | | | Living with – Short term partner (<6 months) | | | | | | | | | | | | | | | | | | | | | | | Living with – Long term partner (>= 6 months) | | | | | | | | | | | | | | | | | | | | | | | | | | | | | With Gharwali/ Madam | | | | | | | | | | | | | | | | | | | | | With the group of sex workers | | | | | | | | | | | | | | | | | | | | | | | With others – Specify | | | | | | | |
| 1.13 | What is the occupation of your husband/partner with whom you are currently living? (use codes)  (Ask if response in 1.12 is 3 or 4 or 5) | | | | | | | | | | | | | | | | | | | | | | | | | | | | | | | | | | | | | | | | | | | | | | | | | | | | | | | | | | | | | | | | | | | | | | | | | | | | | | | | | | | | | | | | | | | | | | | | | | | | | | | | | | | | | | | | | | | | |  | | | | |
| **Code :** Unemployed -1, Driving -2, Daily wages Labor – 3, Self Employed – 4 , Salaried – Pvt - 5 , Salaried – Govt - 6, Seasonal migration-7, Others (Specify) - 77 | | | | | | | | | | | | | | | | | | | | | | | | | | | | | | | | | | | | | | | | | | | | | | | | | | | | | | | | | | | | | | | | | | | | | | | | | | | | | | | | | | | | | | | | | | | | | | | | | | | | | | | | | | | | | | | | | | | | | | | | | | |
| 1.14 | What is the monthly income of your significant partner? (Write in Rupees) | | | | | | | | | | | | | | | | | | | | | | | | | | | | | | | | | | | | | | | | | | | | | | | | | | | | | | | | | | | | | | | | | | | | | | | | | | | | | | | | | | | | | | | | | | | | | | | | | | | | | | | | | | | | | | | | | | | | | **Rs.** | | | | |
| 1.15 | How many dependents do you have? | | | | | | | | | | | | | | | | | | | | | | | | | | | | | | | | | | | | | | | | | | | | | | | | | | | | | | | | | | | | | | | | | |  | | | | | | | | | | | | | | | |  | | | | | | | | | | |  | | | | | | | | | | | |  | | | | | | | | | | | |  | | | | |
| 1.15A | Total number of household members, including you? | | | | | | | | | | | | | | | | | | | | | | | | | | | | | | | | | | | HH members | | | | | | | | | | | | | | | | | | | | | | | | Earning members | | | | | | | | | | | | | | | | | | | | | | |  | | | | | | | | | | |  | | | | | | | | | | | |  | | | | | | | | | | | |  | | | | |
|  |  |  |  |  |  |  |  |  |  |  |  |  |  |  |  |  |  |  |  |  |  |  |  |  |  |  |  |  |  |  |  |  |  |  |  |  | | | | | | | | | | | | | | | | | | | | | | | |  | | | | | | | | | | | | | | | | | | | | | | |  | | | | | | | | | | |  | | | | | | | | | | | |  | | | | | | | | | | | |  | | | | |
| 1.16 | Do you have any children dependent on you? If Yes, provide the details according to birth order | | | | | | | | | | | | | | | | | | | | | | | | | | | | | | | | | | | | | | | | | | | | | | | | | | | | | | | | | | | | | | | | | | Yes | | | | | | | | | | | | | | | | 1 | | | | | | | | | | | No | | | | | | | | | | | | 2 | | | | | | | | | | | | **►1.17** | | | | |
| Birth order | | | | | | Gender.  Male – 1. Female – 2 | | | | Age | | | | | | | Is he/she going to school/College  Yes – 1, No – 2 | | | | | | | | | | | | | | | | | | If Yes, Studying in Which Class / year of College | | | | | | | | | | | | | | | | | | | | | | | | | | | | | | | | | | | | | | | | | | | | | Where is he/she living | | | | | | | | | | | | | | | | | | | Codes :  With Self – 1,  Respondent’s Parents – 2,  Husband -3,  Boarding School – 4,  Relatives – 5,  Others - 77 (Specify) | | | | | | | | | | | | | | | | | | | | | | | |
| Child 1 | | | | | |  | | | |  | | | | | | |  | | | | | | | | | | | | | | | | | |  | | | | | | | | | | | | | | | | | | | | | | | | | | | | | | | | | | | | | | | | | | | | |  | | | | | | | | | | | | | | | | | | |  |  |  |  |  |  |  |  |  |  |  |  |  |  |  |  |  |  |  |  |  |  |  |  |
| Child 2 | | | | | |  | | | |  | | | | | | |  | | | | | | | | | | | | | | | | | |  | | | | | | | | | | | | | | | | | | | | | | | | | | | | | | | | | | | | | | | | | | | | |  | | | | | | | | | | | | | | | | | | |  |  |  |  |  |  |  |  |  |  |  |  |  |  |  |  |  |  |  |  |  |  |  |  |
| Child 3 | | | | | |  | | | |  | | | | | | |  | | | | | | | | | | | | | | | | | |  | | | | | | | | | | | | | | | | | | | | | | | | | | | | | | | | | | | | | | | | | | | | |  | | | | | | | | | | | | | | | | | | |  |  |  |  |  |  |  |  |  |  |  |  |  |  |  |  |  |  |  |  |  |  |  |  |
| Child 4 | | | | | |  | | | |  | | | | | | |  | | | | | | | | | | | | | | | | | |  | | | | | | | | | | | | | | | | | | | | | | | | | | | | | | | | | | | | | | | | | | | | |  | | | | | | | | | | | | | | | | | | |  |  |  |  |  |  |  |  |  |  |  |  |  |  |  |  |  |  |  |  |  |  |  |  |
| 1.17 | Since How many years you are in sex work? Write number of years | | | | | | | | | | | | | | | | | | | | | | | | | | | | | | | | | | | | | | | | | | | | | | | | | | | | | | | | | | | | | | | | | | | | | | | | | | | | | | | | | | | | | | | | | | | | | | | | | | | | | | | | |  | | | | | | | | | | | | | | | | |
| 1.18 | | Please circle months in which they work as sex worker | | | | | | | | | | | | | | 1 | | | | | | 2 | | | | | 3 | | | | | | | | | | | | 4 | | | | | | | 5 | | | | | | | | | | 6 | | | | | | | | | | 7 | | | | | | | | | 8 | | | | | | | | | | | | 9 | | | | | | | | | | | | | | | | 10 | | | | | | | | | 11 | | | | | | | 12 | | | |
|  |  |  |  |  |  |  |  |  |  |  |  |  |  |  |  | Jan | | | | | | Feb | | | | | March | | | | | | | | | | | | April | | | | | | | May | | | | | | | | | | June | | | | | | | | | | July | | | | | | | | | Aug | | | | | | | | | | | | Sept | | | | | | | | | | | | | | | | Oct | | | | | | | | | Nov | | | | | | | Dec | | | |
| 1.19 | | Write the name/s of the towns they work from in the respective month | | | | | | | | | | | | | |  | | | | | |  | | | | |  | | | | | | | | | | | |  | | | | | | |  | | | | | | | | | |  | | | | | | | | | |  | | | | | | | | |  | | | | | | | | | | | |  | | | | | | | | | | | | | | | |  | | | | | | | | |  | | | | | | |  | | | |
| 1.20 | | How often you change the place for sex work? (Circle the relevant, single option) | | | | | | | | | | | | | | | | | | | | | | | | | | | | | | | | | | | | | | | | | | | | | | | | | | | | | | | | | | | | | | | | | | | | | | | | | | | | | | | | | | | | | | | | | | | | | | | | | | | | | | | | | | | | | | | | | | | | | | | | |
| 1 | | 2 | | | | | | | 3 | | | | | | | | | | | | | | | | 4 | | | | | | | | | | | | | | | | | | | | | | | | | | | | | | 5 | | | | | | | | | | | | | | | | | | | | | | | | | | | | | | | | 6 | | | | | | | | | | | | | | | | | | | | | | | | | | | | | | | | | | | |
| Daily | | Weekly | | | | | | | Fortnightly | | | | | | | | | | | | | | | | Once in a month | | | | | | | | | | | | | | | | | | | | | | | | | | | | | | Irregularly | | | | | | | | | | | | | | | | | | | | | | | | | | | | | | | | Never | | | | | | | | | | | | | | | | | | | | | | | | | | | | | | | | | | | |
| 1.21 | | On an average how many paid clients you get in a week? (Write number of clients) | | | | | | | | | | | | | | | | | | | | | | | | | | | | | | | | | | | | | | | | | | | | | | | | | | | | | | | | | | | | | | | | | | | | | | | | | | | | | | | Occasional or New clients | | | | | | | | | | | | | | | | | | | | | | | | | | | | | | | | | | | |  | | | | | |
|  |  |  |  |  |  |  |  |  |  |  |  |  |  |  |  |  |  |  |  |  |  |  |  |  |  |  |  |  |  |  |  |  |  |  |  |  |  |  |  |  |  |  |  |  |  |  |  |  |  |  |  |  |  |  |  |  |  |  |  |  |  |  |  |  |  |  |  |  |  |  |  |  |  |  |  |  |  |  |  |  | Regular clients | | | | | | | | | | | | | | | | | | | | | | | | | | | | | | | | | | | |  | | | | | |
|  |  |  |  |  |  |  |  |  |  |  |  |  |  |  |  |  |  |  |  |  |  |  |  |  |  |  |  |  |  |  |  |  |  |  |  |  |  |  |  |  |  |  |  |  |  |  |  |  |  |  |  |  |  |  |  |  |  |  |  |  |  |  |  |  |  |  |  |  |  |  |  |  |  |  |  |  |  |  |  |  | Total no. of clients | | | | | | | | | | | | | | | | | | | | | | | | | | | | | | | | | | | |  | | | | | |
| Please collect following details | | | | | | | | | | | | | | | | | | | | | | | | Occasional or New clients | | | | | | | | | Regular clients | | | | | | | | | | | | Codes | | | | | | | | | | | | | | | | | | | | | | | | | | | | | | | | | | | | | | | | | | | | | | | | | | | | | | | | | | | | | | | | | | | | | | | | | | | | | |
| A | | | How do you primarily solicit your clients (Use codes) | | | | | | | | | | | | | | | | | | | | |  | | | | | | | | |  | | | | | | | | | | | | At hot spot – **1,** Contact through phone – **2,** Clients will contact – **3,** through network operators/pimps/Brokers – **4,** through Face Book/internet – **5,** They come directly **– 6,** through co sex worker **– 7,** Others **– 8** | | | | | | | | | | | | | | | | | | | | | | | | | | | | | | | | | | | | | | | | | | | | | | | | | | | | | | | | | | | | | | | | | | | | | | | | | | | | | |
| B | | | Where do you primarily solicit clients? | | | | | | | | | | | | | | | | | | | | |  | | | | | | | | |  | | | | | | | | | | | | Home - 1, Rented Room – 2, Lodge/Hotels – 3, Dhaba – 4, Brothel – 5, Bar/Night club – 6, Truck Terminals – 7, Highway/Road – 8, Public place – 9, Massage parlor – 10, Other – 11 | | | | | | | | | | | | | | | | | | | | | | | | | | | | | | | | | | | | | | | | | | | | | | | | | | | | | | | | | | | | | | | | | | | | | | | | | | | | | |
| C | | | On average, how much did you charge a client in the last month? | | | | | | | | | | | | | | | | | | | | |  | | | | | | | | |  | | | | | | | | | | | | ≤ 100 – **1**, 101 to 300 – **2,** 301 to 500 – **3**, 501 to 750 - **4** 751 to 1000- **5,**> 1000 – **6,** | | | | | | | | | | | | | | | | | | | | | | | | | | | | | | | | | | | | | | | | | | | | | | | | | | | | | | | | | | | | | | | | | | | | | | | | | | | | | |
| D | | | What is the minimum amount you charged a client in the last month? | | | | | | | | | | | | | | | | | | | | | Rs. _____ | | | | | | | | | Rs. _____ | | | | | | | | | | | |  | | | | | | | | | | | | | | | | | | | | | | | | | | | | | | | | | | | | | | | | | | | | | | | | | | | | | | | | | | | | | | | | | | | | | | | | | | | | | |
| E | | | What is the maximum amount you charged a client in the last month? | | | | | | | | | | | | | | | | | | | | | Rs. _____ | | | | | | | | | Rs. _____ | | | | | | | | | | | |  | | | | | | | | | | | | | | | | | | | | | | | | | | | | | | | | | | | | | | | | | | | | | | | | | | | | | | | | | | | | | | | | | | | | | | | | | | | | | |
|  | | |  | | | | | | | | | | | | | | | | | | | | | 1.21H. Did you have sex with the following partners in the last 12 months?  Yes 1  No 2 **►1.21JJ** | | | | | | | | | | | | | | | | | | | | 1.21I. How often did you use a condom with the following partners in the last 12 months?  1. Every time; 2. Most of the time, 3. Some times, 4. Never | | | | | | | | | | | | | | | | | | | | | | | | | | | | 1.21J.  Did you use a condom the last time you had sex with the following partners?  Yes 1  No 2 | | | | | | | | | | | | | | | | | | | | | | | | | | | | | | | 1.21JJ. Did you have Anal sex with the following partners in the last 12 months?  Yes 1  No 2 | | | | | | | | | | | | | | | | | | | |
| A. | | | Husband/live-in partner | | | | | | | | | | | | | | | | | | | | |  | | | | | | | | | | | | | | | | | | | |  | | | | | | | | | | | | | | | | | | | | | | | | | | | |  | | | | | | | | | | | | | | | | | | | | | | | | | | | | | | |  | | | | | | | | | | | | | | | | | | | |
| B. | | | Non regular, non-paying partner | | | | | | | | | | | | | | | | | | | | |  | | | | | | | | | | | | | | | | | | | |  | | | | | | | | | | | | | | | | | | | | | | | | | | | |  | | | | | | | | | | | | | | | | | | | | | | | | | | | | | | |  | | | | | | | | | | | | | | | | | | | |
|  | | |  | | | | | | | | | | | | | | | | | | | | | 1.21K. Did you have sex with the following clients in the last month?  Yes 1  No 2**►1.21MM** | | | | | | | | | | | | | | | | | | | | 1.21L. How often did you use a condom with the following clients in the last month?  1. Every time; 2. Most of the time, 3. Some times, 4. never | | | | | | | | | | | | | | | | | | | | | | | | | | | | 1.21M. Did you use a condom the last time you had sex with the following clients?  Yes 1  No 2 | | | | | | | | | | | | | | | | | | | | | | | | | | | | | | | 1.21MM. Did you have Anal sex with the following Clients in the last 12 months?  Yes 1  No 2 | | | | | | | | | | | | | | | | | | | |
| A. | | | Regular clients | | | | | | | | | | | | | | | | | | | | |  | | | | | | | | | | | | | | | | | | | |  | | | | | | | | | | | | | | | | | | | | | | | | | | | |  | | | | | | | | | | | | | | | | | | | | | | | | | | | | | | | | | | | | | | | | | | | | | | | | | | |
| B. | | | Occasional or new clients | | | | | | | | | | | | | | | | | | | | |  | | | | | | | | | | | | | | | | | | | |  | | | | | | | | | | | | | | | | | | | | | | | | | | | |  | | | | | | | | | | | | | | | | | | | | | | | | | | | | | | | | | | | | | | | | | | | | | | | | | | |
| 1.21N | | | In the past 6 months, have you ever faced a situation when you wanted to use condoms but could not do so? | | | | | | | | | | | | | | | | | | | | | | | | | | | | | | | | | | | | | | | | | | | | | | | | | | | | | | | | | | | | | | | | | | | | | Yes  No | | | | | | | | | | | | | | | | | | | | | | | | | | | | | | | | | | | | | | | | | 1  2**►1.22** | | | | | | | | | |
| 1.21O | | | What was the main reason for not using condom? | | | | | | | | | | | | | | | | | | | | Client did not want to use  I did not have a condom with me that time  The PE/ORW could not supply condoms and hence I did not have it  Others (specify) | | | | | | | | | | | | | | | | | | | | | | | | | | | | | | | | | | | | | | | | | | | | | | | | | 1  2  3  9 | | | | | | | | | | | | | | | | | | | | | | | | | | | | | | | | | | | | | | | | |  | | | | | | | | | |
| 1.21P | | | How often did this situation occur in the last six months because the PE/ORW could not supply the required condoms? | | | | | | | | | | | | | | | | | | | | Never  On a few occasions  Half the times  Most of the times  Always | | | | | | | | | | | | | | | | | | | | | | | | | | | | | | | | | | | | | | | | | | | | | | | | | 1  2  3  4  5 | | | | | | | | | | | | | | | | | | | | | | | | | | | | | | | | | | | | | | | | |  | | | | | | | | | |
| 1.22 | | | Do you feel the need for using lubricants? | | | | | | | | | | | | | | | | | | | | | | | | | | Yes | | | | | | | | | | | 1 | | | | | | | | | | | No | | | | | | | | | | | | 2 | | | | | | | | | | | | | | | | Don’t Know | | | | | | | | | | | | | | | | | | | | | | | | | 3 | | | | | | | | | | | | | | | | | | |
| 1.23 | | | Do you use lubricants? | | | | | | | | | | | | | | | | | | | | | | | | | | Yes | | | | | | | | | | | 1 | | | | | | | | | | | No | | | | | | | | | | | | 2 | | | | | | | | | | | | | | | | Don’t Know | | | | | | | | | | | | | | | | | | | | | | | | | 3 | | | | | | | | | | | | | | | | | | |
| 1.24 | | | Other than sex work, are you doing any other work for earning livelihood? | | | | | | | | | | | | | | | | | | | | | | | | | | | | | | | | | | | | | | | | | | | | | | | | | | | | | | | | | | | Yes | | | | | | | | | | | | | | 1 | | | | | | | | | | | | | | | No | | | | | | | | | 2 | | | | | | | | | | | | | | | | | **►1.27** | | | | | |
| 1.25 | | | What work do you do? (Circle the relevant option) | | | | | | | | | | | | | | | | | | | | | | | | | | | | | | | | | | | | | | | | | | | | | | | | | | | | | | | | | | | | | | | | | | | | | | | | | | | | | | | | | | | | | | | | | | | | | | | | | | | | | | | | | | | | | | | | | | | | | | | |
| 1 | | | | | | | 2 | | | | | | | 3 | | | | | | | | | | | | 4 | | | | | | | | | | | | | | | | | | | | | 5 | | | | | | | | | | | | | | | | | | | | | | | | | | | | | | | 6 | | | | | | | | | | | | | | | | | | | | | | | | | | | | | | | | | 77 | | | | | | | | | | | |
| Daily Labor | | | | | | | House maid | | | | | | | Petty shop | | | | | | | | | | | | Private Job | | | | | | | | | | | | | | | | | | | | | Government Job | | | | | | | | | | | | | | | | | | | | | | | | | | | | | | | Street vendor | | | | | | | | | | | | | | | | | | | | | | | | | | | | | | | | | Others – Specify | | | | | | | | | | | |
| 1.26 | | | How much do you earn from this job in month? | | | | | | | | | | | | | | | | | | | | | | | | | | | | | | | | | | | | | | | | | | | | | | | | | | | | | | | | | | | | | | | | | | | | | | | | | | | | | | | | | | | | | | | | | | | | | | | | | | | | Rs/- | | | | | | | | | | | | | | | | | | | |
| 1.27 | | | How much do you earn from sex work in a month? | | | | | | | | | | | | | | | | | | | | | | | | | | | | | | | | | | | | | | | | | | | | | | | | | | | | | | | | | | | | | | | | | | | | | | | | | | | | | | | | | | | | | | | | | | | | | | | | | | | | Rs/- | | | | | | | | | | | | | | | | | | | |
| 1.28 | | | Do you consume ~~a~~lcoholic drinks? | | | | | | | | | | | | | | | | | | | | | | | | | | | | | | | | | | | | | | | | | | Yes  No | | | | | | | | | | | | | | | | | | | | | | | | | | | | | | | | | | 1  2**►1.28B** | | | | | | | | | | | | | | | | | | | | | | | | | | | | | | | | | | | | | | | | | | | |
| 1.28A | | | How often do you consume alcoholic drinks? | | | | | | | | | | | | | | | | | | | | | | | | | | | | | | | | | | | | | | | | | | Some times ( < 3 times a week)  Regularly (>3 times a week)  When client forces | | | | | | | | | | | | | | | | | | | | | | | | | | | | | | | | | | 1  2  3 | | | | | | | | | | | | | | | | | | | | | | | | | | | | | | | | | | | | | | | | | | | |
| 1.28B | | | During the past six months have you suffered from any of these symptoms? | | | | | | | | | | | |  | | | | | | | | | | | | | | | | | | | | | | | | | | | | | | | | | | | | | | | | | | | | | | | | | | | | | | | | | | | | | | | | | | | | | | | | | | Yes | | | | | | | | | | | | | No | | | | | | | |  | | | | | | | | | | | | |
|  |  |  |  |  |  |  |  |  |  |  |  |  |  |  | Genital sore/ulcer | | | | | | | | | | | | | | | | | | | | | | | | | | | | | | | | | | | | | | | | | | | | | | | | | | | | | | | | | | | | | | | | | | | | | | | | | | 1 | | | | | | | | | | | | | 2 | | | | | | | | If no to all in 1.28B, skip to 1.30 | | | | | | | | | | | | |
|  |  |  |  |  |  |  |  |  |  |  |  |  |  |  | Yellowish/greenish discharge from vagina with or without foul smell | | | | | | | | | | | | | | | | | | | | | | | | | | | | | | | | | | | | | | | | | | | | | | | | | | | | | | | | | | | | | | | | | | | | | | | | | | 1 | | | | | | | | | | | | | 2 | | | | | | | |  |  |  |  |  |  |  |  |  |  |  |  |  |
|  |  |  |  |  |  |  |  |  |  |  |  |  |  |  | Lower abdominal pain when not suffering from diarrhea/ dysentery | | | | | | | | | | | | | | | | | | | | | | | | | | | | | | | | | | | | | | | | | | | | | | | | | | | | | | | | | | | | | | | | | | | | | | | | | | 1 | | | | | | | | | | | | | 2 | | | | | | | |  |  |  |  |  |  |  |  |  |  |  |  |  |
| 1.29 | | | Are you suffering from any of these signs or Symptoms NOW? | | | | | | | | | | | |  | | | | | | | | | | | | | | | | | | | | | | | | | | | | | | | | | | | | | | | | | | | | | | | | | | | | | | | | | | | | | | | | | | | | | | | | | | Yes | | | | | | | | | | | | | No | | | | | | | |  | | | | | | | | | | | | |
|  |  |  |  |  |  |  |  |  |  |  |  |  |  |  | Genital sore/ulcer | | | | | | | | | | | | | | | | | | | | | | | | | | | | | | | | | | | | | | | | | | | | | | | | | | | | | | | | | | | | | | | | | | | | | | | | | | 1 | | | | | | | | | | | | | 2 | | | | | | | |  | | | | | | | | | | | | |
|  |  |  |  |  |  |  |  |  |  |  |  |  |  |  | Yellowish/greenish discharge from vagina with or without foul smell | | | | | | | | | | | | | | | | | | | | | | | | | | | | | | | | | | | | | | | | | | | | | | | | | | | | | | | | | | | | | | | | | | | | | | | | | | 1 | | | | | | | | | | | | | 2 | | | | | | | |  | | | | | | | | | | | | |
|  |  |  |  |  |  |  |  |  |  |  |  |  |  |  | Lower abdominal pain when not suffering from diarrhea/ dysentery | | | | | | | | | | | | | | | | | | | | | | | | | | | | | | | | | | | | | | | | | | | | | | | | | | | | | | | | | | | | | | | | | | | | | | | | | | 1 | | | | | | | | | | | | | 2 | | | | | | | |  | | | | | | | | | | | | |
| 1.30 | | | If you suffer from any of the above mentioned symptoms, do you know where do you get treatment?  *Collect the name and address of the clinic/hospital* | | | | | | | | | | | | Not aware | | | | | | | | | | | | | | | | | | | | | | 1 | | | | | | | | | | | | | | | |  | | | | | | | | | | | | | | | | | | | | | | | | | | | | | | | | | | | | | | | | | | | | | | | | | | | | | | | | | | | | | | | | | | | | | |
|  |  |  |  |  |  |  |  |  |  |  |  |  |  |  | Public Hospital / Clinic | | | | | | | | | | | | | | | | | | | | | | 2 | | | | | | | | | | | | | | | |  | | | | | | | | | | | | | | | | | | | | | | | | | | | | | | | | | | | | | | | | | | | | | | | | | | | | | | | | | | | | | | | | | | | | | |
|  |  |  |  |  |  |  |  |  |  |  |  |  |  |  | Private Hospital / Clinic | | | | | | | | | | | | | | | | | | | | | | 3 | | | | | | | | | | | | | | | |  | | | | | | | | | | | | | | | | | | | | | | | | | | | | | | | | | | | | | | | | | | | | | | | | | | | | | | | | | | | | | | | | | | | | | |
|  |  |  |  |  |  |  |  |  |  |  |  |  |  |  | Charitable Hospital | | | | | | | | | | | | | | | | | | | | | | 4 | | | | | | | | | | | | | | | |  | | | | | | | | | | | | | | | | | | | | | | | | | | | | | | | | | | | | | | | | | | | | | | | | | | | | | | | | | | | | | | | | | | | | | |
|  |  |  |  |  |  |  |  |  |  |  |  |  |  |  | TI Clinic | | | | | | | | | | | | | | | | | | | | | | | 5 | | | | | | | | | | | | | | |  | | | | | | | | | | | | | | | | | | | | | | | | | | | | | | | | | | | | | | | | | | | | | | | | | | | | | | | | | | | | | | | | | | | | | |
|  |  |  |  |  |  |  |  |  |  |  |  |  |  |  | Others - Specify __________________________ | | | | | | | | | | | | | | | | | | | | | | | | | | | | | | | | | | | | | | | | | | | | | | | | | | 77 | | | | | | | | | | | | | | | | | | | | | | | | | | | | | | | | | | | | | | | | | | | | | | | | | | | | | | | | | |
| 1.30A | | | In the past 6 months, have you ever faced a situation when you wanted to undergo STI treatment and could not do so? | | | | | | | | | | | | | | | | | | | | | | | | | | | | | | | | | | | | | | | | | | | | | | | | | | | | | | | | | | | | | | Yes  No | | | | | | | | | | | | | | | | | | | | | | | | | | | | | | | 1  2**►1.31** | | | | | | | | | | | | | | | | | | | | | | | | | | |
| 1.30B | | | What was the main reason for not undergoing treatment? | | | | | | | | | | | | | | | | | | | | | | | STI drugs not available at the clinic  Clinic not accessible  Did not have time to visit it  Doctor was not available when I went to visit  I feel discriminated at the clinic and hence do not go for treatment | | | | | | | | | | | | | | | | | | | | | | | | | | | | | | | | | | | | | | | | | | | | | | | | | | | | | | | | | | | | | | | | | | | | | | 1  2  3  4  5 | | | | | | | | | | | | | | | | | | | | | | | | | | |
| 1.30C | | | How often in the past 6 months, were you denied STI treatment because of lack of drugs at the clinic? | | | | | | | | | | | | | | | | | | | | | | | Never  On a few occasions  Half the times  Most of the times  Always | | | | | | | | | | | | | | | | | | | | | | | | | | | | | | | | | | | | | | | | | | | | | | | | | | | | | | | | | | | | | | | | | | | | | | 1  2  3  4  5 | | | | | | | | | | | | | | | | | | | | | | | | | | |
| 1.31 | | | In the last two years how many times have you tested for HIV? | | | | | | | | | | | | | | | | | | | | | | | | | | | | | | | | | | | | | | | | | | | | | | | | | | | | | | | | | | Number …...  Never tested 0 | | | | | | | | | | | | | | | | | | | | | | | | | | | | | | | | | | | | | | | | | | | | | | | If ‘0’ **►1.32** | | | | | | | | | | | | | | |
| 1.31A | | | If you have no problem in sharing, may I know your HIV status? | | | | | | | | | | | | | | | | | | | | | | | | | | | | | | | | | | | | | | | | | | | | | | | | | | | | | | Positive  Negative  Don’t want to share my HIV status | | | | | | | | | | | | | | | | | | | | | | | | | | | | | | | | | | | | | | | | | | | | | | | | | | | 1  2**►1.32**  3**►1.32** | | | | | | | | | | | | | | |
| 1.31B | | | Are you currently on ART? | | | | | | | | | | | | | | | | | | | | | | | | | | | | | | | | | | | | | | | | | | | | | | | | | | | | | | Yes  No | | | | | | | | | | | | | | | | | | | | | | | | | | | | | | | | | | | | | | | | | | | | | | | | | | | 1  2**►1.32** | | | | | | | | | | | | | | |
| 1.31B1 | | | Are you receiving ART medication regularly from ART center? | | | | | | | | | | | | | | | | | | | | | | | | | | | | | | | | | | | | | | | | | | | | | | | | | | | | | | Yes  No | | | | | | | | | | | | | | | | | | | | | | | | | | | | | | | | | | | | | | | | | | | | | | | | | | | 1  2 | | | | | | | | | | | | | | |
| 1.31B2 | | | Are you consuming ART medication regularly as prescribed? | | | | | | | | | | | | | | | | | | | | | | | | | | | | | | | | | | | | | | | | | | | | | | | | | | | | | | Yes  No | | | | | | | | | | | | | | | | | | | | | | | | | | | | | | | | | | | | | | | | | | | | | | | | | | | 1  2 | | | | | | | | | | | | | | |
| 1.31C | | | In the past 6 months, have you ever faced a situation when you wanted to take ART and could not do so? | | | | | | | | | | | | | | | | | | | | | | | | | | | | | | | | | | | | | | | | | | | | | | | | | | | | | | Yes  No | | | | | | | | | | | | | | | | | | | | | | | | | | | | | | | | | | | | | | | | | | | | | | | | | | | 1  2**►1.32** | | | | | | | | | | | | | | |
| 1.31D | | | What was the main reason for not undergoing treatment? | | | | | | | | | | | | | | | | | | | | | | | | | | | | | | | | | | | | | | | | | | | | | | | | | | | | | | Medicines not available at ART center  Migrated to a different area  ART center not accessible  Fear of disclosure of HIV status to others  I feel discriminated at the ART center and hence do not go for treatment  CD4 count is sufficient  Other – (Specify) | | | | | | | | | | | | | | | | | | | | | | | | | | | | | | | | | | | | | | | | | | | | | | | | | | | 1  2  3  4  5  6  9 | | | | | | | | | | | | | | |
| 1.31E | | | How often in the past 6 months, were you denied ART treatment because of lack of drugs at the clinic? | | | | | | | | | | | | | | | | | | | | | | | | | | | | | | | | | | | | | | | | | | | | | | | | | | | | | | Never  On a few occasions  Half the times  Most of the times  Always | | | | | | | | | | | | | | | | | | | | | | | | | | | | | | | | | | | | | | | | | | | | | | | | | | | 1  2  3  4  5 | | | | | | | | | | | | | | |
| 1.32 | | | Do you know the CO (Please share your CO’s Name) | | | | | | | | | | | | | | | | | | | | | | | | | | | | | | | | | | | | | | | | | | | | | | | | | | | | | | Yes | | | | | | | | | | | | | | | | | | | | | 1 | | | | | | | | | | | | | No | | | | | | | | | | | | | | | | | 2 **►2.1** | | | | | | | | | | | | | | |
| 1.33 | | | Are you a registered member of a community organization (CO)? | | | | | | | | | | | | | | | | | | | | | | | | | | | | | | | | | | | | | | | | | | | | | | | | | | | | | | Yes | | | | | | | | | | | | | | | | | | | | | 1 | | | | | | | | | | | | | No | | | | | | | | | | | | | | | | | 2 **►1.38** | | | | | | | | | | | | | | |
| 1.34 | | | If Yes, Since when did you become a member? (Write number of months and years since registered) | | | | | | | | | | | | | | | | | | | | | | | | | | | | | | | | | | | | | | | | | | | | | | | | | | | | | | | | | | | | | | | | | | | | | | | | | | | | | | | | | | | | | | | | | | | | | | | | | | | | | | | | | Months _____ | | | | | | | | | | | | Years | | |
| 1.35 | | | Have you ever paid annual membership fee? | | | | | | | | | | | | | | | | | | | | | | | | | | | | | | | | | | | | | | | | | | | | | | | | | | | | | | | | Yes | | | | | | | | | | | | | | | | | | | 1 | | | | | | | | | | | | | | | | | | | No | | | | | | | | | | | 2 **►1.38** | | | | | | | | | | | | | | |
| 1.36 | | | If Yes, How much annual membership fee have you paid? | | | | | | | | | | | | | | | | | | | | | | | | | | | | | | | | | | | | | | | | | | | | | | | | | | | | | | | | | | | | | | | | | | | | | | | | | | | | | | | | | | | | | | | | | | | | | | | | | | | | | | | | |  | | | | | | | | | | | | | | |
| 1.37 | | | When did you paid last annual membership fee? | | | | | | | | | | | | | | | | | | | | | | | | | | | | | | | | | | | | | | | | | | | | | | | | | | | | | | | | | | | | | | | | | | | | | | | | | | | | | | | | | | | | | | | | | | | | | | | | | | | | | | | | | MM/YYYY | | | | | | | | | | | | | | |
| 1.38 | | | Do you share the information shared with you by the field workers with other KPs? | | | | | | | | | | | | | | | | | | | | | | | | | | | | | | | | | | | | | | | | | | | | | | | | | | | | | | | | | | | | | | | | | | | | | | | Yes | | | | | | | | | | | | | | | | | | | 1 | | | | | | | | | | | | | | | | | | No | | | | | | | | | | 0 | |
| 1.39 | | | Are you a member of some SHG/CBG/CIG/Aastha Gath? | | | | | | | | | | | | | | | | | | | | | | | | | | | | | | | | | | | | | | | | | | | | | | | | | | | | | | | | Yes | | | | | | | | | | | | | | | | | 1 | | | | | | | | | | | | | | | | | | | | | | | | No | | | | | | | | | | | | | | | | | 0 **►1.41** | | | | | |
| 1.40 | | | If yes, what is the name of the group? | | | | | | | | | | | | | | | | | | | | | | | | | | | | | | | | | | | | | | | | | | | | | | | | | | | | | | | |  | | | | | | | | | | | | | | | | | | | | | | | | | | | | | | | | | | | | | | | | | | | | | | | | | | | | | | | | | | | | | | | |
| 1.41 | | | How much you trust the CO’s to resolve your issues? (Circle the code) | | | | | | | | | | | | | | | | | | | | | | | | | | | | | | | | | | | | | | | | | | | | | | | | | | | | | | | | No trust – 1, Some trust – 2, Fully trust – 3 | | | | | | | | | | | | | | | | | | | | | | | | | | | | | | | | | | | | | | | | | | | | | | | | | | | | | | | | | | | | | | | |
| 1.42 | | | How frequently do you interact with the CO? ( Specify the code) | | | | | | | | | | | | | | | | | | | | | | | | | | | | | | | | | | | | | | | | | | | | | | | | | | | | | | | | Every day – **1**, Once a week -**2,** Once every two weeks – **3,** Once a month – **4** Other – specify – **5** | | | | | | | | | | | | | | | | | | | | | | | | | | | | | | | | | | | | | | | | | | | | | | | | | | | | | | | | | | | | | | | |
| 1.43 | | | Can you mention 3 leaders in your CO who you respect and listen to? | | | | | | | | | | | | | | | | | | | | | | | | | | | | | | | | | | | | | | | | | | | | | | | | | | | | | | |  | | | | | | | | | | | | | | | | | | | | | | | |  | | | | | | | | | | | | | | | | | | | | | | | | | | | | | | |  | | | | | | | | | |
| 1.44 | | | Currently are you handling any positions of the CO? | | | | | | | | | | | | | | | | | | | | | | | | | | | | | | | | | | | | | | Yes | | | | | | | | | | | | 1 | | | | | | | | | No | | | | | | | | | | | | | | | | 2 | | | | | | | | | | | | | | | | | | | | | | | | | | | | | | | | | | | | | | | | | | | | |
| 1.45 | | | Have you handled any positions of the CO in the past? | | | | | | | | | | | | | | | | | | | | | | | | | | | | | | | | | | | | | | Yes | | | | | | | | | | | | 1 | | | | | | | | | No | | | | | | | | | | | | | | | | 2 | | | | | | | | | | | | | | | | | | | | | | | | | | | | | | | | | | | | | | | | | | | | |
| 1.46 | | | Do you want to take a leadership Role in CO in future? | | | | | | | | | | | | | | | | | | | | | | | | | | | | | | | | | | | | | | Yes | | | | | | | | | | | | 1 | | | | | | | | | No | | | | | | | | | | | | | | | | 2 | | | | | | | | | | | | | | | | | | | | | | | | | | | | | | | | | | | | | | | | | | | | |
| 1.47 | | | If Yes, What Role would you like to play? (please specify) | | | | | | | | | | | | | | | | | | | | | | | | | | | | | | | | | | | | | | | | | | | | | | | | | |  | | | | | | | | | | | | | | | | | | | | | | | | | | | | | | | | | | | | | | | | | | | | | | | | | | | | | | | | | | | | | | | | | | | | | |
| 1.48 | | | Can you tell us three services you received from the CO during last six months | | | | | | | | | | | | | | | | | | | | | | | | | | | | | | | | | | | | | | | | | | | | | | | | | | | | | | | | | | | | | | | | |  | | | | | | | | | | | | | | | | | | | |  | | | | | | | | | | | | | | | | | | | | | | | | |  | | | | | | | | | |
| 1.49 | | | Can you tell us three things /services you provided or contributed to the CO? | | | | | | | | | | | | | | | | | | | | | | | | | | | | | | | | | | | | | | | | | | | | | | | | | | | | | | | | | | | | | | | | |  | | | | | | | | | | | | | | | | | |  | | | | | | | | | | | | | | | | | | | | | | | | | | |  | | | | | | | | | |
| 1.50 | | | What are the 3 words (feelings) that come to your mind when you think of the CO? | | | | | | | | | | | | | | | | | | | | | | | | | | | | | | | | | | | | | | | | | | | | | | | | | | | | | | | | | | | | | | | | |  | | | | | | | | | | | | | | | | | |  | | | | | | | | | | | | | | | | | | | | | | | | | | |  | | | | | | | | | |
| 1.51 | | | What would you lose if the CO did not exist? | | | | | | | | | | | | | | | | | | | | | | | | | | | | | | | | | | | | | | | | | | | | | | | | | | | | | | | | | | | | | | | | | | |  | | | | | | | | | | | | | | | | | | | | | | | | | | | | | | | | | | | | | | | | | | | | | | | | | | | | |
| 1.52 | | | If you were managing the CO, what is the one thing that you would change? | | | | | | | | | | | | | | | | | | | | | | | | | | | | | | | | | | | | | | | | | | | | | | | | | | | | | | | | | | | | | | | | |  | | | | | | | | | | | | | | | | | | | | | | | | | | | | | | | | | | | | | | | | | | | | | | | | | | | | | | |
| 1.53 | | | | How do you feel about your CO? Why? | | | | | | | | | | | | | | | | | | | | | | | | | | Proud | | | | | | | | | | | | | 1 | | | | | | | Disappointed | | | | | | | | | | | | | | | | | | 2 | | | | | Why? | | | | | | | | | | | | | | | | | | | | | | | | | | | | | | | | | | | | | | | | | | | | | | | | |  |
| 1.54 | | | | | Which of the following phones do you use? | | | | | | | | | | | | | | | | | | | | | | | | | | | Touch screen | | | | | | | | | | | | | | | | | | | | | | 1 | | | | | | | | | | | | | | | | | | | | | | | Button phones | | | | | | | | | | | | | | | | | | | | | | | | | | | | | | 2 | | | | | | | | | | | | | | | |

| ***Section 2 : Access to services - Safety, Security and Justice*** | | | | | | | | | | | | | | | | | | |
| --- | --- | --- | --- | --- | --- | --- | --- | --- | --- | --- | --- | --- | --- | --- | --- | --- | --- | --- |
| 2.1 | Do you know forms of violence | | | Yes | | 1 | | 2.2 | Have you attended any session on legal education | | | | | | Yes | | 1 | |
|  |  |  |  | No | | 2 | |  |  |  |  |  |  |  | No | | 2 | |
| 2.3 | What techniques are used for preventing violence?  (record verbatim) | | | | | | |  | | | | | | | | | | |
|  | 1. In the last six months have you faced any of the following? | | B  Yes -1 No-2 | | C. If Yes,  By Whom?  (Use codes) | | D. If yes, Number of incidents happened in the last 6 months? | | | E. What do you feel about it?  It is normal (can’t do anything) – 1  It is abnormal – 2 (needs to be addressed) | F. Did you report the last incident to anybody?  **Yes-1, No-2**  **►H** | G. If Yes, Whom did you Report?  Uses Code  **►I** | H. If No, Why did you not Report?  Uses Code | | | I. If, Yes, did you receive the justice?  **Yes-1**  **No-2** | | |
| 2.4 | Physical Violence (hurt, hit, slapped, pushed, kicked, punched, choked, or burnt..etc) | | 1  2**►2.5** | |  | |  | | |  |  |  |  | | |  | | |
| 2.5 | Sexual Violence (forced to have sex by anyone when you didn’t want to?) | | 1  2**►2.6** | |  | |  | | |  |  |  |  | | |  | | |
| 2.6 | Abuse, called names etc (Blackmailed, scolded in public….etc) | | 1  2**►2.7** | |  | |  | | |  |  |  |  | | |  | | |
| **C -Codes for faced violence by:** Stranger -1, Client -2, Boyfriend/Partner -3, Husband-4, Pimps/Broker/Mediator - 5, Police - 6, Goons-7, Other Sex worker-8, Madam/ Gharwali – 9, Family ( Other than husband)-10, Others (Specify ___________________) – 77 | | | | | | | | | | | | | | | | | | |
| **G – Codes for violence reported to:** CO Leaders – 1, CO staff – 2, Crisis Response Team – 3, Fellow Sex Worker – 4, Pimp/Madam – 5, Police – 6, Don’t remember – 7, CO help line-8, Other help line-9, Others (Specify ________________________________) – 77 | | | | | | | | | | | | | | | | | | |
| **H – Codes for reasons of not reporting:** Fear of disclosure – 1, Do not know whom to report -2, Don’t know what to do – 3, I was advised not to disclose-4, Not needed – 5, Others (Specify ____________________________) – 77 | | | | | | | | | | | | | | | | | | |
| **2.7** | | According to you, what kind of change has there been in the trends of violence towards you in the past 12 months? Use codes | | | | | | | | | | | | No Change | | | | 1 |
|  |  |  |  |  |  |  |  |  |  |  |  |  |  | Increasing | | | | 2 |
|  |  |  |  |  |  |  |  |  |  |  |  |  |  | Decreasing | | | | 3 |

| ***Section 3: Access to services - Social Protection*** | | | | | | | | | | | | | | | | | | | | | |
| --- | --- | --- | --- | --- | --- | --- | --- | --- | --- | --- | --- | --- | --- | --- | --- | --- | --- | --- | --- | --- | --- |
| SN | Civic Identities | | | | A. Do you have these **Yes-1, No-2 ►C** | | B. If yes, since when (Write in no of months) **►E** | | C. If no, have you applied?  **Yes-1, No-2►Next row** | | | D. If yes, when did you apply ( write in the no of months) | | E. Who helped you to get/apply  (Use codes) | | | F. Has your application for this id ever been rejected?  **Yes-1, No-2** | | | G. Reasons for rejection? | |
| 3.1 | Ration/BPL Card | | | |  | |  | |  | | |  | |  | | | 1  2 **►3.2** | | |  | |
| 3.2 | Voter ID | | | |  | |  | |  | | |  | |  | | | 1  2 **►3.3** | | |  | |
| 3.3 | Aadhar Card | | | |  | |  | |  | | |  | |  | | | 1  2 **►3.4** | | |  | |
| 3.4 | PAN Card | | | |  | |  | |  | | |  | |  | | | 1  2 **►3.5** | | |  | |
| 3.5 | Gas Connection | | | |  | |  | |  | | |  | |  | | | 1  2 **►3.6** | | |  | |
| 3.6 | Nativity, caste certificate | | | |  | |  | |  | | |  | |  | | | 1  2 **►3.7** | | |  | |
| Codes: E: No One-1; Family Members-2; Living Partner-3; CO leader/member-4; SP Champion-5; Sex workers ( non CO members)-6, Living Partner-7, NGO Staff-8; Govt. Officials-9; CO Staff-10; Others-99  Codes G: A. Insufficient supporting documents, B. Not eligible, C. Beyond deadline, D. Incomplete form, E. Quota over, F. Asking for facilitation fee, G. Stigma, H. Recommendation required, Z. Other (specify) | | | | | | | | | | | | | | | | | | | | | |
| SN | | Scheme: (List the top Specific schemes prioritized in the state) | | A. Are you aware about this scheme**Yes-1, No-2 ►next row** | | B.If Yes, have you benefitted **Yes-1, No-2►D** | | C. If yes, When did you get the benefit?(No. of months before) **►F** | | D. If No, Have you applied **Yes-1,**  **No-2** **►Next row** | | | E. If Yes, When did you apply? ( Write in No. of months before ) | | F. Who helped you to get/apply (Use codes) | | | G. Has your application for this id ever been rejected?  **Yes-1, No-2** | | | H. Reasons for rejection? |
| 3.7 | |  | |  | |  | |  | |  | | |  | |  | | | 1  2 **►3.8** | | |  |
| 3.8 | |  | |  | |  | |  | |  | | |  | |  | | | 1  2 **►3.9** | | |  |
| 3.9 | |  | |  | |  | |  | |  | | |  | |  | | | 1  2 **►3.10** | | |  |
| 3.10 | |  | |  | |  | |  | |  | | |  | |  | | | 1  2 **►3.11** | | |  |
| 3.11 | |  | |  | |  | |  | |  | | |  | |  | | | 1  2 **►3.12** | | |  |
| 3.12 | |  | |  | |  | |  | |  | | |  | |  | | | 1  2 **►3.13** | | |  |
| 3.13 | |  | |  | |  | |  | |  | | |  | |  | | | 1  2 **►3.14** | | |  |
| 3.14 | |  | |  | |  | |  | |  | | |  | |  | | | 1  2 **►3.15** | | |  |
| 3.15 | |  | |  | |  | |  | |  | | |  | |  | | | 1  2 **►3.16** | | |  |
| 3.16 | |  | |  | |  | |  | |  | | |  | |  | | | 1  2 **►3.17** | | |  |
| Codes : F : No one – 1, Family members – 2, Living Partner – 3, CO Leader/ Member – 4, SP Champion -5, Sex workers (non-member of CO) – 6 , NGO staff – 8, Govt Officials – 9, CO Staff – 10, Others - 77  Codes H: A. Insufficient supporting documents, B. Not eligible, C. Beyond deadline, D. Incomplete form, E. Quota over, F. Asking for facilitation fee, G. Stigma, H. Recommendation required, Z. Other (specify) | | | | | | | | | | | | | | | | | | | | | |
| 3.17 | | | How do you feel about the process? | | | | | | | | Insulted/Dissatisfied-1 | | | | | Satisfied -2 | | | No feelings-3 | | |

| ***Section 4: Access to services - Financial Security*** | | | | | | | | | | | | | | | | | | | | | | | |
| --- | --- | --- | --- | --- | --- | --- | --- | --- | --- | --- | --- | --- | --- | --- | --- | --- | --- | --- | --- | --- | --- | --- | --- |
| 4.1 | | | Do you have a Savings Account in Bank or Post Office? | | | | Yes | | 1 | | No | 2 **► 4.3** | | 4.1.0 | How many accounts do you have? | | | | | | |  | |
| **Enter the details of the 2 most used bank accounts in case, the number of bank accounts are more than 2.** | | | | | | | | | | | | | | | | | | | | | | | |
|  | In which Bank/Post Office? | | | Did someone from the CO help you in opening the account? | | | | Since how many months you had this account? ( Write number of months) | | | Do you own the following?  1.Cheque Book  2. ATM/Debit card  3. Online/Mobile banking | | | | How many transactions have you done in the past quarter? | | | | When was the last transaction made?  MM/YY | | | | |
| 4.1.1 |  | | |  | | | |  | | |  | | | |  | | | |  | | | | |
| 4.1.2 |  | | |  | | | |  | | |  | | | |  | | | |  | | | | |
| 4.1.4 | Did you have to share your ATM PIN with your partner/someone else | | | | | | | | | | | | | Yes | | 1 | | | No | | | 2 | |
| 4.1.5 | Do you need permission of your partner/ someone else for withdrawing money from bank? | | | | | | | | | | | | | | | | | Yes | | 1 | | No | 2 |
| 4.2 | | A.Have you invested in the following saving schemes Use Codes : Yes – 1, No-2  **If 2, ►next financial product/row** | | | | B.If yes, Where did you invest. Use Codes  **Bank -1**  **Post Office – 2** | | | | C.If yes, When did you invest  (Number of months before) | | | D. Who helped you to invest? (Use codes) | | | | E. Has your application for this ever been rejected?  **Yes-1, No-2** | | | | F. Reasons for rejection? | | |
| 4.2.1 | | Recurring Deposit (RD) | | |  |  | | | |  | | |  | | | | 1  2 **►4.2.2** | | | |  | | |
| 4. 2.2 | | Fixed Deposit (FD) | | |  |  | | | |  | | |  | | | | 1  2 **►4.2.3** | | | |  | | |
| 4.2.3 | | Public Provident Fund (PPF) | | |  |  | | | |  | | |  | | | | 1  2 **►4.2.4** | | | |  | | |
| 4.2.4 | | National Saving Certificate (NSC)/KisanVikasPatra (KVP) | | |  |  | | | |  | | |  | | | | 1  2 **►4.3** | | | |  | | |
| Codes : D : No one – 1, Family members – 2, Living Partner – 3, CO Leader/ Member – 4, Community Champion -5, Sex workers (non-member of CO) – 6 , NGO staff – 7, Govt Officials – 8, CO Staff – 9, Others – 77  Codes F: A. Insufficient supporting documents, B. Not eligible, C. Beyond deadline, D. Incomplete form, E. Quota over, F. Asking for facilitation fee, G. Stigma, H. Recommendation required, Z. Other (specify) | | | | | | | | | | | | | | | | | | | | | | | |
| \| 4.3 \| \| \| 1. Are you doing savings in the following sources?   Use Codes : Yes – 1, No-2 **If 2, ►next source/row** \| \| \| \| \| \| \| \| \| B. If yes, Since when  (Number of months before) \| \| \| \| \| C. Who helped you to Save? (Use codes) \| \| \| \| \| \| \| \| \| \| --- \| --- \| --- \| --- \| --- \| --- \| --- \| --- \| --- \| --- \| --- \| --- \| --- \| --- \| --- \| --- \| --- \| --- \| --- \| --- \| --- \| --- \| --- \| --- \| --- \| --- \| \| 4.3.1 \| \| \| Self-help group \| \| \| \| \|  \| \| \| \|  \| \| \| \| \|  \| \| \| \| \| \| \| \| \| \| 4.3.2 \| \| \| Cooperatives \| \| \| \| \|  \| \| \| \|  \| \| \| \| \|  \| \| \| \| \| \| \| \| \| \| 4.3.3 \| \| \| Shares / Mutual fund / Company Bond \| \| \| \| \|  \| \| \| \|  \| \| \| \| \|  \| \| \| \| \| \| \| \| \| \| 4.3.4 \| \| \| Chit fund/ Bishi \| \| \| \| \|  \| \| \| \|  \| \| \| \| \|  \| \| \| \| \| \| \| \| \| \| 4.3.5 \| \| \| Friends/ relatives/ family member \| \| \| \| \|  \| \| \| \|  \| \| \| \| \|  \| \| \| \| \| \| \| \| \| \| 4.3.6 \| \| \| Money lender \| \| \| \| \|  \| \| \| \|  \| \| \| \| \|  \| \| \| \| \| \| \| \| \| \| 4.3.7 \| \| \| Bank/Post office \| \| \| \| \|  \| \| \| \|  \| \| \| \| \|  \| \| \| \| \| \| \| \| \| \| 4.3.8 \| \| \| Others (Specify__________________) \| \| \| \| \|  \| \| \| \|  \| \| \| \| \|  \| \| \| \| \| \| \| \| \| \| Codes : C : No one – 1, Family members – 2, Living Partner – 3, CO Leader/ Member – 4, Community Champion -5, Sex workers (non-member of CO) – 6 , NGO staff – 7, Govt Officials – 8, CO Staff – 9, Others – 77 \| \| \| \| \| \| \| \| \| \| \| \| \| \| \| \| \| \| \| \| \| \| \| \| \| \| \| 4.4 \| \| 1. Have you invested in the following products?   Use Codes : Yes – 1, No-2 **If 2,►next investment/row** \| \| \| \| \| \| \| \| \| \| \| B. If yes, Since when  (Number of months before) \| \| \| \| \| \| \| C. Who helped you to Save (Use codes) \| \| \| \| \| \| \| 4.4.1 \| \| Gold \| \| \| \| \| \| \| \|  \| \| \|  \| \| \| \| \| \| \|  \| \| \| \| \| \| \| 4.4.2 \| \| Land \| \| \| \| \| \| \| \|  \| \| \|  \| \| \| \| \| \| \|  \| \| \| \| \| \| \| 4.4.3 \| \| Residential Plot /House \| \| \| \| \| \| \| \|  \| \| \|  \| \| \| \| \| \| \|  \| \| \| \| \| \| \| 4.4.4 \| \| Business \| \| \| \| \| \| \| \|  \| \| \|  \| \| \| \| \| \| \|  \| \| \| \| \| \| \| 4.4.5 \| \| Any other (Specify) \| \| \| \| \| \| \| \|  \| \| \|  \| \| \| \| \| \| \|  \| \| \| \| \| \| \| 4.5 \| \| Have you invested in the following products? Use Codes Yes – 1, No-2 **If 2,►next investment/row** \| \| \| \| \| If yes, Since when  (Number of months before) \| \| \| \| \| \| \| When did you pay the last premium? (No of months back), if not known enter 99 \| \| \| \| \| \| \| \| C. Who helped you to Save (Use codes) \| \| \| \| \| 4.5.1 \| \| Life insurance \| \| \|  \| \|  \| \| \| \| \| \| \|  \| \| \| \| \| \| \| \|  \| \| \| \| \| 4.5.2 \| \| Health insurance \| \| \|  \| \|  \| \| \| \| \| \| \|  \| \| \| \| \| \| \| \|  \| \| \| \| \| 4.5.3 \| \| Accidental Insurance \| \| \|  \| \|  \| \| \| \| \| \| \|  \| \| \| \| \| \| \| \|  \| \| \| \| \| Codes : C : No one – 1, Family members – 2, Living Partner – 3, CO Leader/ Member – 4, SP Champion -5, Sex workers (non-member of CO) – 6 , NGO staff – 7, Govt Officials – 8, CO Staff – 9, Others – 77 \| \| \| \| \| \| \| \| \| \| \| \| \| \| \| \| \| \| \| \| \| \| \| \| \| \| \| 4.6 \| \| In the past one year have you taken any loan? \| \| \| \| \| \| \| \| \| \| \| \| \| \| Yes \| \| \| 1 \| \| No \| \| 2 \| \| **►4.6.2** \| \| 4.6.1 \| \| If Yes, How many loans? Collect number of loans taken from all sources (Formal & Informal) \| \| \| \| \| \| \| \| \| \| \| \| \| \| \| \| \| \|  \| \| \| \| \| \| \| Collect details for all the loans \| \| \| \| \| \| \| \| \| \| \| \| \| \| \| \| \| \| \| \| \| \| \| \| \| \| \|  \| A. Source of loan  **Use Codes** \| \| \| B. What was the purpose of loan?  **Use Codes** \| \| C. Loan amount  (Write the amount)  Rs. \| \| \| D. What is the rate of interest?  Per Annum \| \| E. Have you pledged/mortgaged anything to get the loan?  Yes -1 No -2 \| \| \| \| F. If Yes, provide the details?  Use codes  Gold -1, Mortgaged property – 2  Other – 3 \| \| \| G. Duration for Repayment (Write in Months) \| \| \| \| \| \| H. Are you Repaying the loan regularly?  Yes -1  No-2 \| \| \| A \|  \| \| \|  \| \|  \| \| \|  \| \|  \| \| \| \|  \| \| \|  \| \| \| \| \| \|  \| \| \| B \|  \| \| \|  \| \|  \| \| \|  \| \|  \| \| \| \|  \| \| \|  \| \| \| \| \| \|  \| \| \| C \|  \| \| \|  \| \|  \| \| \|  \| \|  \| \| \| \|  \| \| \|  \| \| \| \| \| \|  \| \| \| **A. Source of Loan :** Bank -1, MFI, – 2, Cooperative Bank - 3, SHG – 4, Money Lender – 5, Friend & Relatives – 6,NBFC-7, Brothel owners-8, Others – 77 (Specify) \| \| \| \| \| \| \| \| \| \| \| \| \| \| \| \| \| \| \| \| \| \| \| \| \| \| \| **B. Purpose of Loan :** Personal ill-health - 1 Husband/partner ill-health - 2 Children’s ill-health - 3 Children’s education - 4 To repay earlier loan - 5 To bribe - 6, To start the business – 7, To purchase the asset-8, For marriage – 9, Festival – 10, House repair -11, Others (specify) - 77 \| \| \| \| \| \| \| \| \| \| \| \| \| \| \| \| \| \| \| \| \| \| \| \| \| \| \| \| 4.6.2 \| Have you ever defaulted on some loan? \| \| \| \| \| \| Yes \| 1 \| No \| \| 2 \| **►4.6.6** \| \| --- \| --- \| --- \| --- \| --- \| --- \| --- \| --- \| --- \| --- \| --- \| --- \| --- \| \| 4.6.3 \| If yes, what was the reason for the last default? ( Multiple options) \| High interest rate-1 \| \| No income-2 \| Emergency situation-3 \| \| Other(Specify)-77 \| \| \| \| \| \| \| 4.6.4 \| What was the amount when you had last defaulted \| \|  \| \| \| \| \| \| \| \| \| \| \| 4.6.5 \| Since how long has the loan been ou tstanding? \| \| <6 months-1 \| \| \| 6 months-1 year \| \| \| \| >1 year \| \| \| \| \| 4.6.6 \| Were you ever denied loan from any institution or individuals? \| \| \| \| \| \| \| Yes \| \| \| 1 \| \|  \| \| \| No \| \| \| 2 \| \| **►4.6.8** \| \| --- \| --- \| --- \| --- \| --- \| --- \| --- \| --- \| --- \| --- \| --- \| --- \| --- \| --- \| --- \| --- \| --- \| --- \| --- \| --- \| --- \| --- \| \| 4.6.7 \| What are the reasons? Circle the relevant (Multiple answers possible) \| \| \| \| \| \| \| \| \| \| \| \| \| \| \| \| \| \| \| \| \| \| 1 \| \| 2 \| \| \| 3 \| \| \| \| 4 \| \| \| \| \| \| 5 \| \| \| \| \| \| \| \| No Guarantee \| \| No documents \| \| \| W s not eligible \| \| \| \| Ill treated \| \| \| \| \| \| Other (Specify) \| \| \| \| \| \| \| \| 4.6.8 \| Have you faced any financial crisis in the last six months? (You were in urgent need of money but did not get it or you did not have enough money to pay the loan/installment) \| \| \| \| \| \| \| \| \| \| \| \| \| \| Yes \| \| 1 \| \| \|  \| \| \| No \| \| 2 \| \| \| **►5.1** \| \| \| 4.6.9 \| I f Yes, What was the crisis? Use Codes \| \| \| \| \| \| \| \| \| \| \| \| \| \|  \| \| \| \| \| \| \| \| Codes : Inability to pay for any day to day needs or any commitments – 1, Losing money from informal sources – 2, Sudden surge in financial needs (could happen due to any sudden reduction in income due to health, closure of brothel, raid, etc.; disasters; losing a family member; loan repayments) – 3 \| \| \| \| \| \| \| \| \| \| \| \| \| \| \| \| \| \| \| \| \| \| \| 4.70 \| How did you cope with the situation? (Multiple options possible) \| \| \| \| \| \| \| \| \| \| \| \| \| \|  \| \| \| \| \| \| \| \| Resorted to sex without condom \| \| \| 1 \| Entertained more clients \| \| 2 \| Defaulted on loan \| \| \| 3 \| \| Borrowed from informal sources \| \| \| \| \| \| 4 \| \| \| \| \| sold assets \| \| \| 5 \| Claimed Insurance \| \| 6 \| Others \| \| \| 77 \| \| Specify \| \|  \| \| \| \| \| \| \| \| \| \| \| \| \| \| \| \| \| \| \| \| \| \| \| \| \| \| \| \| \| \| \| \| \| \| \| \| \| \| \| \| \| \| \| \| \| \| \| | | | | | | | | | | | | | | | | | | | | | | | |

| ***Section 5 :Food Insufficiency*** | | | |
| --- | --- | --- | --- |
| 5.1 | In the last one month, did you or other adults in your household ever not eat for a whole day because there wasn’t enough money for food | Yes | 1 |
|  |  | No | 2 |
| 5.2 | In the last one month, did you or other adults in your household ever not eat at night because there wasn’t enough money for food | Yes | 1 |
|  |  | No | 2 |
| 5.3 | How did you cope with the situation? Use codes |  | |
| Code : Resorted to sex without condom-1, Entertained more clients – 2, Defaulted on loan – 3, Borrowed from informal sources- 4, sold assets – 5, Other – 77 | | | |

| Sr No | Questions | Response for self | Response for children |
| --- | --- | --- | --- |
| 5.4 | The food that we bought just didn't last, and we didn’t have money to get more. | 1. Was that often  2. Sometimes  3. Never true for you in the last 6 months | NA |
| 5.5 | In the last 6 months, did you or other adults in your household ever cut the size of your meals or skip meals because there wasn’t enough money for food | 1. Yes  2. No**►5.7** | 1. Yes  2. No**►5.7** |
| 5.6 | How often did this happen? | 1.Almost every month  2. Some months but not every month,  3. One or two months | 1.Almost every month  2. Some months but not every month,  3. One or two months |
| 5.7 | In the last 6 months, did you ever eat less than you felt you should because there wasn’t enough money to buy food? | 1.Yes  2. No | 1.Yes  2. No |
| 5.8 | In the last 6 months, were you ever hungry but didn’t eat because you couldn’t afford enough food? | 1.Yes  2.No | 1.Yes  2.No |

| ***Section 6: Reproductive Health*** | | | | | | | | | | |
| --- | --- | --- | --- | --- | --- | --- | --- | --- | --- | --- |
| 6.1 | Do you use pads or cloths during menstrual periods? | Pads | | | 1 |  | Cloths | 2 |  | |
| 6.2 | Do you use any family Planning method? | Yes | | | 1 |  | No | 2 | **►6.4** | |
| 6.3 | Which method are you using currently? | | | | | | Sterilization | | 1 | |
|  |  |  |  |  |  |  | Oral Pills | | 2 | |
|  |  |  |  |  |  |  | Condoms | | 3 | |
|  |  |  |  |  |  |  | IUD (Copper T) | | 4 | |
|  |  |  |  |  |  |  | Injectables | | 5 | |
|  |  |  |  |  |  |  | Others (Specify) | | 77 | |
|  |  |  |  |  |  |  |  | |  | |
| 6.4 | Do you want to have a child and unable to conceive one? | | Yes | 1 | | | No | 2 | | **►7.0** |
| 6.5 | Have you taken any treatment for this? | | Yes | 1 | | | No | 2 | | |

| ***Section 7: Birth History*** | | | | |
| --- | --- | --- | --- | --- |
| 7.0 | Have you ever become pregnant | Yes  No | 1  2 | **►7.7** |
| 7.1 | At what age did you have your last pregnancy? | Age (in years)_____ |  |  |
| 7.2 | When you were pregnant last time, did you want to become pregnant then, did you want to wait until later, or did you not want to have any children at all? | Then  Wanted to wait until Later  Did not want | 1  2  3 |  |
| 7.3 | How many total pregnancies have you had in your lifetime? |  | Number____ |  |
| 7.4 | How many births have you had in your lifetime? | 1. Live births 2. Still births | Number____  Number____ |  |
| 7.5 | How many abortions have you had in your lifetime? | 1. Spontaneous 2. Induced | Number____  Number____ | If “0”**►7.7** |
| 7.6 | How many induced abortions have you experienced in the past 24months? |  | Number____ |  |
| 7.7 | In the last 12 months, how many times have you used emergency contraceptive pills? |  | Number____ |  |
| 7.8 | In the past 6 months, was there a time community members came together because of a problem that affected all or some of the sex worker? | Yes  No | 1  2 |  |

| ***Section 8. MENTAL HEALTH*** | | | | | |
| --- | --- | --- | --- | --- | --- |
| **DEPRESSION (CES-D SCALE)**  Below is a list of some ways you may have felt or behaved. Please indicate how often you have felt this way during the last week. Please only provide one answer to each question | | | | | |
|  | **During the past week:** | Rarely or none of the time (less than 1 day) | Some or a little of the time (1-2 days) | Occasionally or a moderate amount of time (3-4 days) | Most or all of the time (5-7 days) |
| 8.1 | I was bothered by things that usually don't bother me | 1 | 2 | 3 | 4 |
| 8.2 | I had trouble keeping my mind on what I was doing | 1 | 2 | 3 | 4 |
| 8.3 | I felt depressed. | 1 | 2 | 3 | 4 |
| 8.4 | I felt that everything I did was an effort | 1 | 2 | 3 | 4 |
| 8.5 | I felt hopeful about the future. | 1 | 2 | 3 | 4 |
| 8.6 | I felt fearful | 1 | 2 | 3 | 4 |
| 8.7 | My sleep was restless | 1 | 2 | 3 | 4 |
| 8.8 | I was happy | 1 | 2 | 3 | 4 |
| 8.9 | I felt lonely | 1 | 2 | 3 | 4 |
| 8.10 | I could not do the things I should be doing | 1 | 2 | 3 | 4 |

| ***Section 9: HIV Preventions services*** | | | | |
| --- | --- | --- | --- | --- |
| 9.1 | Did you receive information on STI/HIV/AIDS from a peer educator or an outreach worker from the targeted intervention (TI) program in the last one year? | Yes  No | 1  2 | **►9.3** |
| 9.2 | How many times in the past one month were you contacted in the field by a peer/worker from  the TI program to give you information? | Number_____ |  |  |
| 9.3 | Did you receive condoms from the peer educator or outreach workers of the targeted intervention (TI) program in the last one year? | Yes  No | 1  2 | **►9.5** |
| 9.4 | In the past one month, how often were you given condoms by a peer/worker from the TI program? | Every day  More than once a week  Once a week  Fortnightly  Once a month  Don’t remember | 1  2  3  4  5  8 |  |
| 9.5 | Did you access check up and counselling services for STI from the targeted intervention (TI) program in the last one year? | Yes  No | 1  2 | **►9.7** |
| 9.6 | How many times have you visited the TI for problems like abnormal/white vaginal discharge or genital ulcers or lower abdominal pain in the last six months OR since you first knew about it? (if LESS than six months)? | Number of times  Don’t know | 98 |  |
| 9.7 | Did you receive free medicines for STIs from the targeted intervention (TI) program in the last one year? | Yes  No | 1  2 |  |
| 9.8 | Were you referred to other services (STI clinic, ICTC etc.) by the targeted intervention (TI) program in the last one year? | Yes  No | 1  2 |  |

**Section 9A: Utilization of Mobile phones/internet**

|  | Have you ever used cell phone to receive information/services related; | Yes | No |  |
| --- | --- | --- | --- | --- |
| 9.9a | HIV prevention services | 1 | 2 |  |
| 9.9b | CO related outreach services | 1 | 2 |  |
| 9.9c | Soliciting clients | 1 | 2 |  |
| 9.9d | To deal with police | 1 | 2 |  |
| 9.9e | To communicate with other FSWs | 1 | 2 |  |
|  | Have you ever used internet/social networking applications to receive information/services related; | |  |  |
| 9.10a | HIV prevention services | 1 | 2 |  |
| 9.10b | CO related outreach services | 1 | 2 |  |
| 9.10c | Soliciting clients | 1 | 2 |  |
| 9.10d | To deal with police | 1 | 2 |  |
| 9.10e | To communicate with other FSWs | 1 | 2 |  |

| ***Section 10: Costing*** | | | | | | | |
| --- | --- | --- | --- | --- | --- | --- | --- |
| 10.1 Entitlements/ Schemes  (If >6, collect information on the last 6) | 10.2  How many visits did you have to make for receiving/claiming the entitlement/ scheme the last time? | **10.3.U.**  On average, how much time did you spend in receiving the entitlement/ scheme in each visit, including travel time? (UNIT)  Hours  Days  Weeks  Months  (If they report in minutes, enter 1 hour) | **10.3.V.**  On average, how much time did you spend in receiving the entitlement/ scheme in each visit, including travel time? (VALUE)  _ _ | 10.4  How much money did you spend on travel for each visit you made to get the entitlement/ scheme? | 10.5  How many days did you take off from work to get the entitlement/ scheme? | 10.6  What was the quantum of monetary benefit of the entitlement/ scheme? | 10.7  Did you have to pay either in cash or kind to anyone in the process of getting this entitlement/scheme? |
| 1. |  |  |  |  |  |  |  |
| 2. |  |  |  |  |  |  |  |
| 3. |  |  |  |  |  |  |  |
| 4. |  |  |  |  |  |  |  |
| 5. |  |  |  |  |  |  |  |
| 6. |  |  |  |  |  |  |  |

|  |  | 10.8  The last time you experienced the following, how many days did you have to take off from work? | 10.9  The last time you experienced the following, how many days did it take for you to get back to your normal life? | 10.10  The last time you experienced the following, how much money did you spend on medical, legal, police, counselling and other services? |
| --- | --- | --- | --- | --- |
|  | Physical Violence (hurt, hit, slapped, pushed, kicked, punched, choked, or burnt etc.) | Number of days _____ | Number of days _____ | INR________ |
|  | Sexual Violence (forced to have sex by anyone when you didn’t want to?) | Number of days _____ | Number of days _____ | INR________ |
|  | Abuse, called names etc. (Blackmailed, scolded in public….etc.) | Number of days _____ | Number of days _____ | INR________ |
